# Synthesis, biological evaluation and clinical trials of Cereblon-based PROTACs

**DOI:** 10.1038/s42004-025-01598-9

**Published:** 2025-07-29

**Authors:** André T. S. Vicente, Sara P. S. P. Moura, Jorge A. R. Salvador

**Affiliations:** 1 Laboratory of Pharmaceutical Chemistry, Faculty of Pharmacy, University of Coimbra, Coimbra, Portugal; 2 Center for Neuroscience and Cell Biology, University of Coimbra, Coimbra, Portugal; 3 Center for Innovative Biomedicine and Biotechnology (CIBB), University of Coimbra, Coimbra, Portugal

**Keywords:** Diversity-oriented synthesis, Pharmacology, Drug discovery and development

## Abstract

PROteolysis-Targeting Chimeras (PROTACs) are an emerging class of molecules capable of inducing a forced approximation between a protein of interest (POI) and an E3 ligase enzyme (e.g., Cereblon), leading to the degradation of the POI by the cell’s own machinery. Although in early stages of development, PROTACs’ unique mechanisms of action offer a novel therapeutic strategy, which has attracted growing interest worldwide. Cereblon-based PROTACs are the most studied class of PROTACs and have been actively researched in recent years for the treatment of different diseases, from cancer to neurological disorders, with some of them already in clinical trials. In this review, we provide a comprehensive and critical analysis covering the recent advances, potential challenges and future prospects regarding the design and synthesis, as well as pre- and clinical evaluation of cereblon-based PROTACs. By integrating insights from drug discovery and development, a broad yet in-depth discussion is given to guide future research on cereblon-based PROTACs.

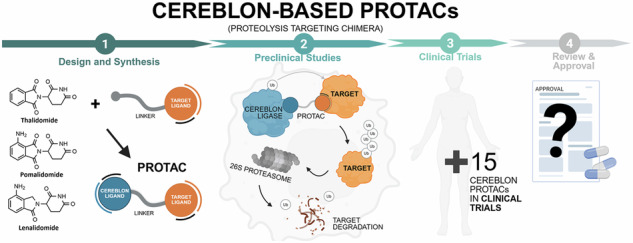

## Abstract (web summary)

PROteolysis-Targeting Chimeras (PROTACs) represent a promising therapeutic approach by facilitating the degradation of target proteins via E3 ligase enzymes. Here, the authors analyze recent advances and challenges in cereblon-based PROTACs from synthesis to clinical trials, offering insights to guide future development in cereblon-based targeted protein degradation.

## Introduction

Involved in almost all biological and pathological mechanisms, whether inside or outside cells, proteins are one of the most important building blocks of life^1^. Throughout their entire life cycle, proteins are subject to tight regulation at the pre-, co- and post-translational levels, which is critical for the maintenance of proteostasis^2,3^. The coordinated and precise interaction between the ubiquitin-proteasome system (UPS), the protein chaperone system, and the autophagy-lysosome system (ALS) results in a dynamic endogenous protein quality control system that is essential to ensure the protein homeostasis^4–6^. Focusing on the UPS, this is the main proteolysis pathway for short-lived, damaged, oxidized, misfolded, or abnormal proteins^7^. It is also involved in the normal turnover process of proteins that have fulfilled their functions^8,9^. Simplistically, the UPS can be divided into two subsystems: the ubiquitination system, which is responsible for the addition of ubiquitin moieties to target proteins, and the proteasome system, which is responsible for the recognition and degradation of poly-ubiquitinated proteins^10,11^. Ubiquitin-mediated protein degradation by the UPS was discovered in the 1980s, and begins with the poly-ubiquitination of the target, that is, with the addition to the protein to be degraded of several ubiquitin units - a 76-aminoacid regulatory protein of globular conformation^12,13^. However, the ubiquitination state of a protein also defines, for example, its cellular location, or its recognition by signaling or regulatory complexes^14,15^. From the sequential activity of ubiquitin-activating enzymes (E1), ubiquitin–conjugating enzymes (E2), and ubiquitin protein ligases (E3), ubiquitin is catalytically added to the target in an ATP-dependent process^16^. Target specificity is ensured by direct binding between the E3 ligase, which recognizes the substrate and transfers the ubiquitin units to its surface^17^. The ATP-dependent proteolytic complex, called the 26S proteasome, then recognizes the polyubiquitin protein and degrades it into small peptide fragments^18^.

**Fig. 1:**
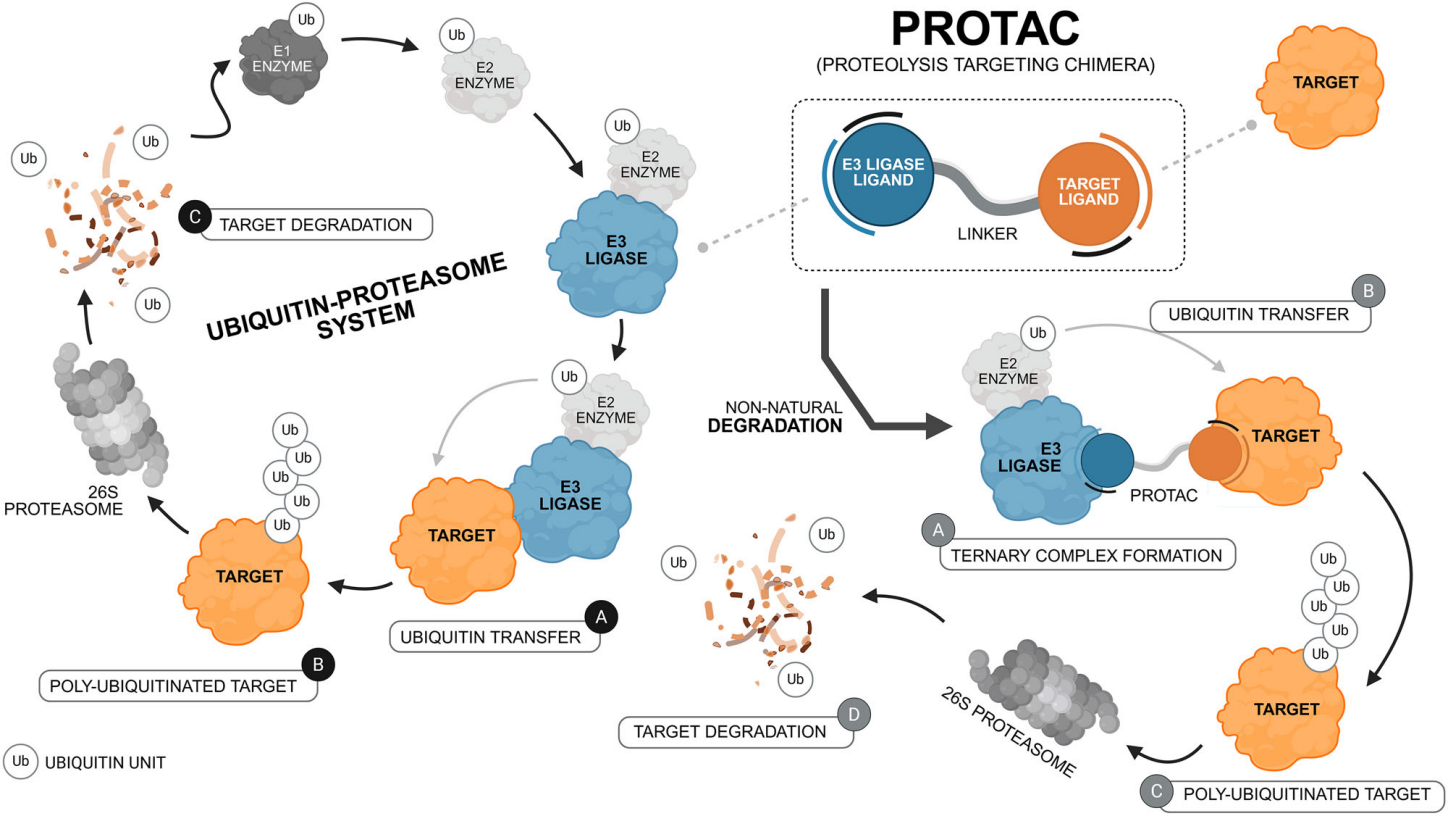
Mechanism of action of PROteolysis-TArgeting Chimeras (PROTACs). In the Ubiquitin-Proteasome System (UPS), the process of ubiquitination begins when the ubiquitin protein (Ub) is activated by the ubiquitin-activating E1 enzyme. The activated Ub is then transferred to the ubiquitin-conjugating enzyme E2. Afterwards, the E3 ubiquitin ligase connects both to the target protein and to the E2-Ub complex, enabling the attachment of ubiquitin molecules to the target. Once tagged with a polyubiquitin chain, the target protein is recognized and degraded by the 26S proteasome. PROTACs are heterobifunctional molecules containing a target protein ligand at one end, and an E3 ligase ligand capable of recruiting an E3 ligase enzyme from the UPS to the other end, with a linker establishing the connection between the two ligands. Within the cell, PROTAC promotes the simultaneous and artificial binding of the target protein to an E3 ligase, forming a stable ternary complex. This leads to the target poly-ubiquitination and subsequent non-natural degradation by the UPS.

In 2001, the drug discovery paradigm was completely revolutionized by the pioneering work of Crews and Deshaies, who published the first compound capable of directing the UPS against a protein of interest (POI), thereby forcing its non-natural degradation (Fig. 1)^19^. This successful proof-of-concept study opened the doors to the PROteolysis-TArgeting Chimeras (PROTACs) development. PROTACs are heterobifunctional molecules containing a pharmacophoric moiety capable of binding to the POI (target ligand moiety (TLM)) at one end and an E3 ligase binding moiety (ELM) capable of recruiting an E3 binding enzyme from the UPS at the other end^20^. The connection between these two ligands (TLM and ELM) is established by a chemical linker^21^. Mechanistically, once inside the cell, PROTAC forms a stable ternary complex resulting from its simultaneous binding to the POI and a specific E3 ligase^22^. By forcing proximity between the target protein and the E3 ligase, PROTAC induces proximity-driven polyubiquitination of the POI, i.e., the proximity forced by the PROTAC results in the transfer of the ubiquitin units from the E3 ligase to the POI^23^. After polyubiquitination, the target protein dissociates from PROTAC and follows the normal UPS pathway, where it is recognized by the 26S proteasome and degraded into small peptide fragments^24^. Afterwards, the PROTAC is free to form a new stable ternary complex with another POI, thus exhibiting an advantageous catalytic mechanism of action (event-driven mechanism)^25^. Twenty-five years after the publication of the first PROTAC, these are included within the Targeted Protein Degradation (TPD) field, which encompasses all innovative strategies aimed at chemically-induced protein degradation, known generically as degraders^26^. However, unquestionably PROTACs are the most popular within the TPD field^27^.

The PROTACs boom was sparked by several causes. Firstly, due to its revolutionary mechanism of action, in which the suppression of the biological effect is obtained through the degradation of the therapeutic target and protein aggregates, using the cell’s own machinery^20^. Secondly, due to the ability to suppress all functions of a given protein, as well as “undruggable proteins” (e.g., transcription factors or scaffold proteins), since PROTAC does not need to bind to a specific site on the target protein^22^. Third, due to preclinical and clinical outcomes that have demonstrated that PROTACs have the potential to overcome many of the challenges observed with the use of small molecular inhibitors (SMIs), such as the development of resistance or serious side effects^27^. The consolidation of the therapeutic potential of PROTACs has led to an intensification of the race to discover better PROTACs^28^.

In general, PROTACs are classified according to the E3 ligase recruited. To date, of the more than 600 different E3 ligases identified in human cells, the vast majority of reported PROTACs, including those in clinical studies, recruit the cereblon E3 ligase^29^. The second most commonly recruited E3 ligase by PROTACs is the Von Hippel-Lindau (VHL) E3 ligase^29^. To a lesser extent, PROTACs also recruit the inhibitor of apoptosis protein (IAP) and the mouse double minute ligases (MDM2)^29^. Recently, the activity of new PROTACs that recruit other E3 ligases, such as the Kelch-like ECH-associated protein 1 (KEAP1) E3 ligase, the DDB1-CUL4A-associated factor 16 (DCAF16) E3 ligase and the RING finger protein 114 (RNF114) E3 ligase, has been investigated^30–32^. There are numerous articles demonstrating that cereblon-based PROTACs can rapidly, potently and highly selectively degrade, both in vitro and in vivo, a huge number of different proteins associated with the development of various diseases, including cancer, neurodegenerative diseases, immune diseases, cardiovascular diseases and viral infections^33,34^.

Undoubtedly, cereblon-based PROTACs are frontrunners in the PROTAC field, and have aroused global interest from the pharmaceutical industry and academia^35^. Therefore, this comprehensive review aims to provide an up-to-date critical overview on cereblon-based PROTACs, covering the main aspects related to the chemical synthesis of cereblon ligands derivatives, the chemical ligation of these derivatives to the linkers, and the construction of the final cereblon PROTAC. Furthermore, relevant and recent examples of novel cereblon-based PROTACs will be presented and the main results obtained from their biological evaluation will be analyzed. In this review, potential challenges and future perspectives of PROTACs that recruit the cereblon E3 ligase will also be discussed.

## Cereblon—structure and function

In 2004, Joseph J. Higgins described the cereblon protein for the first time, after mapping the deletion and mutation of its gene in a patient with autosomal recessive non-syndromic mental retardation^36^. Cereblon designation derives from its high expression in the cerebellum, its importance in mental development, as well as its highly conserved ATP-dependent Lon domain^37^. Among the various domains of cereblon, the drug-binding region called cereblon domain stands out, which is responsible for recognizing and binding to endogenous target proteins, as well as specific immunomodulatory imide drugs (IMiDs), with emphasis on thalidomide, lenalidomide and pomalidomide^4^.

Cereblon encodes a 442 amino acid protein, with a molecular weight of 51 kDa, which is widely expressed in all tissues^4,37,38^. Cereblon functions as a substrate specificity receptor/substrate recognition protein within the Cullin 4-really interesting new gene (RING) E3 ubiquitin ligase CRL4^CRBN^ complex^39^. With approximately 400 different members, the CRL4 subfamily is the largest group of cullin RING E3 ligases^38^. The CRL4^CRBN^ complex is composed of cereblon, which interacts with damaged DNA-binding protein 1 (DDB1), Cullin4 (Cul4A/4B), and regulator of cullins 1 (RBX1)^40^. In this complex, the cereblon is the unit responsible for recognizing and binding to target proteins for subsequent ubiquitination, while the DDB1 protein functions as a support linking cereblon to the Cullin4A/4B proteins, which in turn serve as molecular scaffolds^33,41^. Although the intracellular location of Cereblon depends on the cell type in question, in general, it is distributed throughout the cell, including in the nucleus, given that the N-terminal region of cereblon can function as an important signal for nuclear localization^4,37^.

Over the last few decades, different targets of cereblon have been identified, concluding that the targets vary according to the tissue and cellular location of this E3 ligase^42^. Cereblon has gained prominence as a potential therapeutic target in several diseases since, by interacting with several proteins involved in multiple downstream signaling pathways, it ends up controlling a wide range of different physiological processes crucial for normal cellular functioning^4,37^. For example, although not all of its targets are known, many are involved in the regulation of ion channels, immune system, and metabolism^38^. Some of its targets have also been implicated in the development of many diseases, including cerebral, cardiovascular, and oncological diseases^38^. Recently, the ubiquitination-independent functions of cereblon have been studied, which include its function as a transcription repressor, chaperone protein, and scaffold protein^37^.

## Cereblon ligands

Molecular mechanistic studies with the controversial drug thalidomide led to the discovery of cereblon’s ligands^43^. Developed in the 1950s by the German pharmaceutical company Chemie Grünenthal, thalidomide was initially administered as a sedative-hypnotic drug, widely prescribed at the time to treat morning sickness in pregnant women^21,37^. However, a few years later, it was withdrawn from the market due to its severe teratogenicity (>10,000 cases of phocomelia birth defects worldwide) in what became known as the thalidomide tragedy, one of the most devastating in the pharmaceutical industry history^44,45^.

After a few decades, studies showed that thalidomide and its structural analogs have anti-inflammatory, anti-angiogenic and anti-myeloma properties, thus leading to their designation as IMiDs^37,43^. However, it was only in 2010 that Ito’s group demonstrated that thalidomide binds to cereblon E3 ligase^46^. Additional studies have demonstrated that the biologicals effects of thalidomide and IMiDs result from their binding to cereblon, which culminates in the ubiquitination and degradation of neo-substrates^47^. For example, lenalidomide and pomalidomide lead to the degradation of the neo-substrates Ikaros (family zinc finger protein-1, IKZF1) and Aiolos (family zinc finger protein-3, IKZF3) transcriptional factors, thereby exhibiting an anti-myeloma activity^48,49^.

**Fig. 2:**
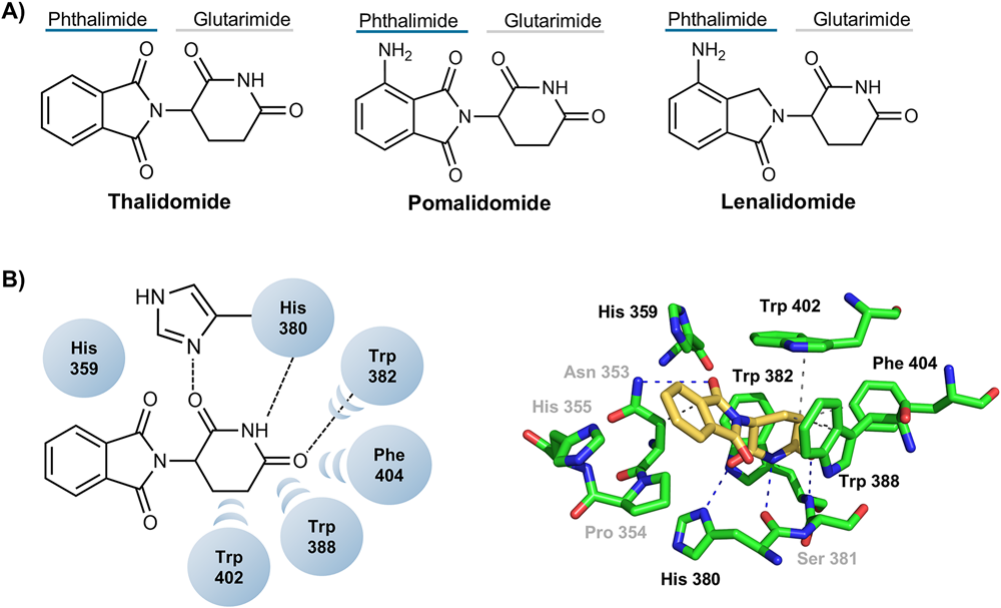
Cereblon ligands. **A** Chemical structure of immunomodulatory drugs (IMiDs): thalidomide, pomalidomide and lenalidomide. **B** Interactions between aminoacids residues of cereblon E3 ligase and thalidomide.

Structurally, the cereblon E3 ligase ligands have a phthalimide unit linked to a glutarimide unit moiety (Fig. 2)^50^. Studies by Fischer et al. to characterize the structure of the cereblon complex and its binding to IMiDs showed that thalidomide, pomalidomide and lenalidomide bind very similarly to cereblon protein^51^. More specifically, in the phthalimide part, the phenyl group is solvent-exposed, while one of the carbonyl groups forms the water-mediated hydrogen bond with His359^21^. The glutarimide moiety occupies a conserved hydrophobic pocket on the C-terminal domain of cereblon, also known as the cereblon domain, with the carbonyl groups and intervening amide interacting with His380 and Trp382 via hydrogen bonding^51^. The remaining carbon atoms of the glutarimide ring interact with the amino acids Trp382, Trp388, Trp402, and Phe404 through van der Waals bonds^51^. The structural analysis of thalidomide and its structural analogs pomalidomide and lenalidomide reveals the presence of a chiral center, which is known to influence the binding affinity to cereblon protein. However, as these compounds racemize under physiological conditions, they are administered as racemic mixtures^52^. In the following schemes, stereochemical information is provided wherever possible. If this does not occur, it is either because the compounds exist in the form of an isomeric mixture, or because such information is not reported in the literature.

Following the discovery of the binding capacity of IMiDs drugs with cereblon E3 ligase, the focus in this area over the last few years has been on the synthesis and study of new structural analogs of IMiDs with the aim of obtaining new and better building blocks for the construction of cereblon-recruiting PROTACs^43,53^.

## Cereblon-based PROTACs—from synthesis to biological evaluation

At the moment, cereblon-based PROTACs are the frontrunners in the PROTACs field. PROTACs recruiting the cereblon E3 ligase have successfully induced the forced degradation of more than 60 different proteins, associated with the development of a wide range of disorders, from cancer to neurological, immunological and viral diseases, with successful examples already in clinical trials^54–58^.

The study of thalidomide chemistry led to the differentiated functionalization of the phthalimide ring, allowing the production of different cereblon ligand libraries for incorporation into the construction of novel cereblon-based PROTACs (Table 1)^59^.

**Table 1:** Summary of chemical syntheses to obtain thalidomide derivatives


Substituent (X =)	Linker Connectivity	Reaction name	Conditions	Product
OH	R-X	Alkylation	Base, Δ	
NH_2_	R-COCl	Acylation	Base, Δ	
F	R_1_-NH-R_2_	S_*N*_Ar	Base, Δ	
Br	Ar-B(OR)_2_	Suzuki coupling	Pd(dppf)Cl_2_, base, Δ	
I	H_2_C = C-R	Heck coupling	Pd_2_(dba)_3_, HP(*t*-Bu)_3_BF_4_, Cy_2_NMe, base, Δ	
Br, I	R-C ≡ CH	Sonogashira coupling	Pd(PPh_3_)_2_Cl_2_, CuI, base, Δ	
OH	R-OH	Mitsunobu	TPP, DIAD	
NH_2_	R-SH	Photoredox sulfuration	Ru(bpy)_3_Cl_2_, TsOH, *t-*BuONO	

To date, cereblon-based PROTACs are the most commonly reported in both the scientific literature and patents, due to the advantages presented by the incorporation of cereblon ligands, compared to the incorporation of ligands of other E3 ligases. Therefore, the success of cereblon-based PROTACs is due to a set of key factors: Firstly, the most commonly used cereblon ligands are IMiDs, as they exhibit highly specific, strong and biophysically validated E3 ligase binding affinity^43^; Secondly, cereblon ligands have desirable physicochemical properties, including low molecular weight, lipophilicity, solubility, chemical and metabolic stability and the absence of pan-assay interference compound (PAINS) warnings^60^. It should be noted that these characteristics are favorable for obtaining PROTACs with satisfactory oral bioavailability; Thirdly, the cereblon ligands present a faithfully characterized structural information about their binding mode to cereblon protein^51^; Fourthly, cereblon ligands are versatile and easily modifiable^61^; Fifthly, cereblon E3 ligase is expressed in multiple tissues, enabling cereblon-based PROTACs to treat various diseases^62^. On the other hand, VHL ligands, although also easily modifiable, are large and lipophilic molecules, which can lead to solubility and permeability problems in PROTACs^63^. Currently available MDM2 ligands may undesirably interfere with the p53 cycle, causing serious adverse effects^64^. Regarding the ligands of E3 ligases less recruited by PROTACs, such as KEAP1 or DCAF, the existing ligands are still scarce, with low affinities and little studied. Therefore, the result of the desirable properties obtained with the incorporation of cereblon ligands in the construction of PROTACs, together with the excellent therapeutic results published in recent years, obtained by forced induction of the degradation of a specific POI by cereblon-based PROTACs in vitro, ex vivo and in vivo, and for different types of diseases, make this type of PROTACs the leaders in the field of TPD. Consequently, the race to discover promising PROTACs recruiting the cereblon E3 ligase has been fierce in both academia and big pharma, as reflected by the dramatic increase in the number of scientific papers and patents filed in recent years^65,66^.

Although a wide range of cereblon ligands that can be used to construct PROTACs are currently reported, most of cereblon-recruiting PROTACs results from attaching different types of linkers to pomalidomide, thalidomide and lenalidomide derivatives. More recently, the incorporation of 5-phthalimide substituted derivatives and novel non-phthalimide cereblon binders have been reported.

**Fig. 3:**
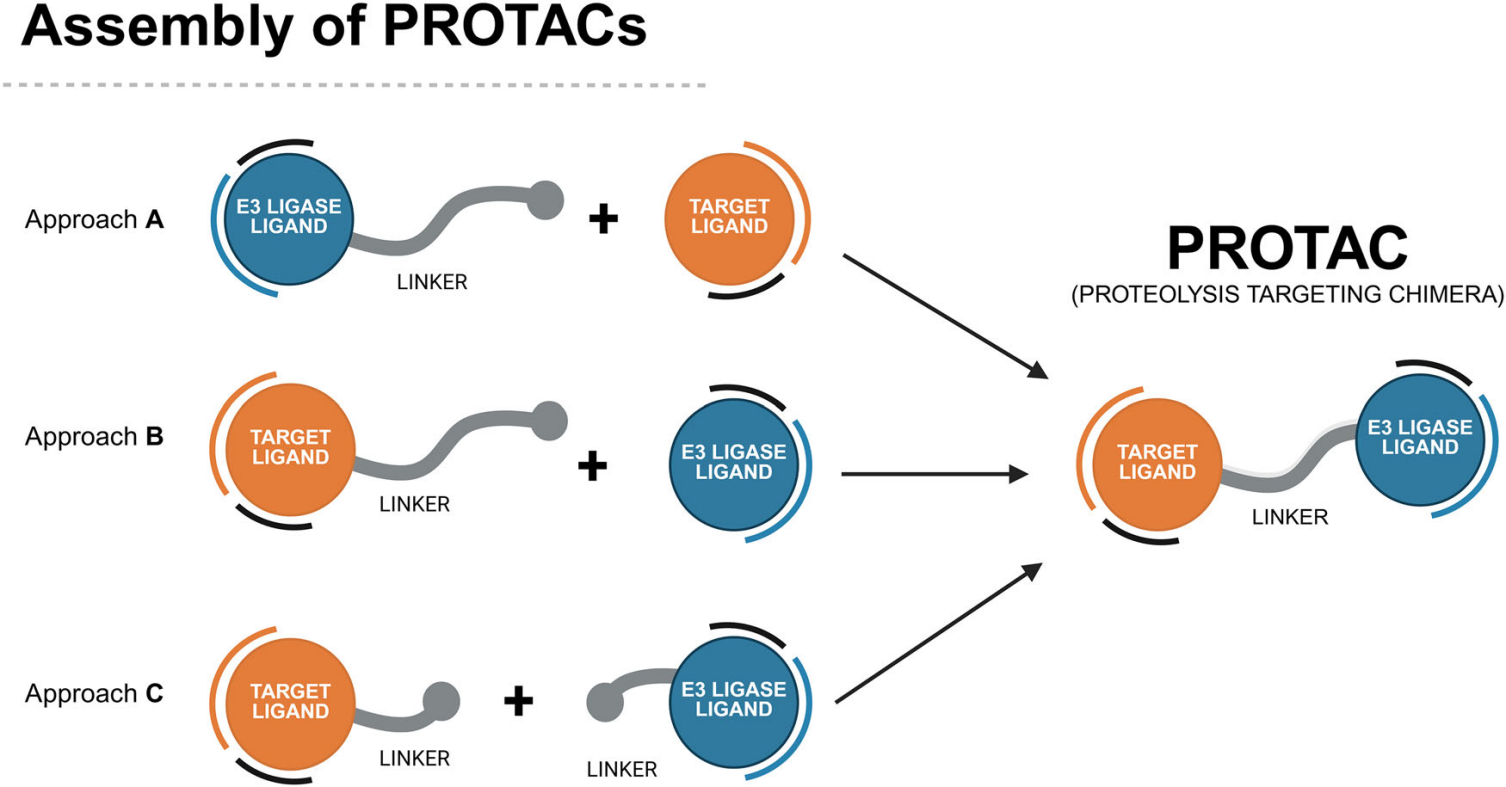
Chemical assembly of cereblon-based PROTACs. Three conventional approaches are used: (Approach A) coupling the cereblon ligand to the linker first, followed by attachment of the target ligand; (Approach B) coupling the linker to the target ligand first, then attaching the cereblon ligand; and (Approach C) connecting two smaller linker fragments separately to the target and cereblon ligands, followed by joining the two halves to form the complete PROTAC molecule.

Regarding the synthetic pathway of cereblon-based PROTACs, there are conventionally three approaches to assemble the different building blocks (i.e., target ligand, linker and cereblon ligand) (Fig. 3):

- First bind the cereblon ligand to the desired linker, and then bind it to the target ligand (Approach A);
- First bind the linker to the target ligand, and then bind it to the cereblon ligand (Approach B);
- Attaching two smaller linker fragments separately to the target and cereblon ligands, then joining the two halves together (Approach C).

In each of the above approaches, it is possible to modify the warhead (i.e., target and cereblon ligands) to facilitate or optimize linker conjugation. The choice of the best approach for the construction of cereblon-based PROTACs depends on factors such as the availability and cost of the building blocks, or the number of steps and purification processes required to obtain the final compound.

For clarity and organizational purposes, this review article categorizes cereblon-based PROTACs according to the cereblon ligand, and further subdivides them based on the type of bond used for linker attachment. In each section, a critical and up-to-date review is provided, covering not only the design and synthetic strategies of PROTACs, but also a detailed study of the chemical synthesis of key building blocks and their attachment to linkers. This review also covers important aspects related to the biological evaluation of cereblon-based PROTACs, with relevant examples published in recent years.

### Pomalidomide-based PROTACs

Pomalidomide-based ligands can generally be synthesized via two different routes. One route is via condensation between a glutarimide ring unit with the 3-fluorophthalic anhydride **(1)**, followed by a nucleophilic aromatic substitution (S_N_Ar) reaction with a linker whose terminus is a primary or secondary amine^61^. Another route is via condensation of the glutarimide ring unit with the 3-nitrophthalic anhydride, with consequent reduction of the nitro group to an amine group, thus producing pomalidomide^64^.

In general, the current synthesis methods for pomalidomide-based PROTACs are low yielding and produce undesirable by-products^61,64^.

#### Pomalidomide-based PROTACs from 3-fluorophtalic anhydride

One of the most widely used strategies for linker attachment in PROTACs is via S_N_Ar reactions with fluorinated derivatives of thalidomide.

**Scheme 1:**
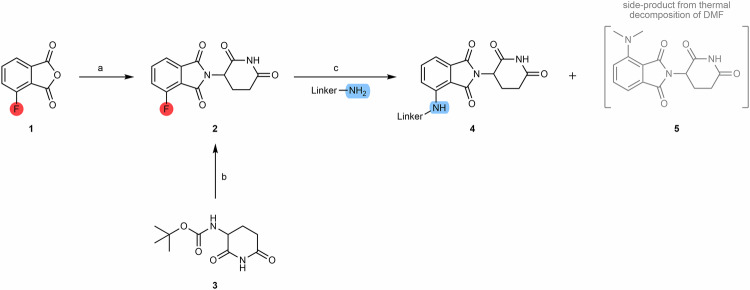
Chemical synthesis of pomalidomide-based PROTACs from 3-fluorophtalic anhydride. Reagents and conditions: **a**_1_ 3-aminopiperidine-2,6-dionehydrochloride, triethylamine, THF, 80 °C; **a**_2_ 3-aminopiperidine-2,6-dionehydrochloride, sodium acetate, acetic acid, 140 °C, 12 h; **b** **1**, sodium acetate, acetic acid, reflux, 6 h; **c** DIPEA, DMF or DMSO, 90 °C to 130 °C, 6 to 16 h (depending on the type of linker).

When the starting compound is 4-fluorothalidomide **(2)**, it is possible to synthesize alkylated pomalidomide derivatives **(4)** by S_N_Ar reactions between 4-fluorothalidomide and linkers with primary or secondary amine end groups, in the presence of N,N-diisopropylethylamine (DIPEA) and using dimethylformamide (DMF) or dimethyl sulfoxide (DMSO) as solvent, at high temperatures (>90 °C) (Scheme 1)^67,68^. Although DMF is a common solvent used in this type of reaction, the high temperatures associated with the presence of tertiary amines (e.g., DIPEA) can result in thermal decomposition of the DMF into a dimethylamine by-product. In turn, dimethylamine can react with 4-fluorothalidomide and form the undesirable product 4-(dimethylamino)-thalidomide **(5)**^69–71^. Replacement of DMF with DMSO, dioxane or dimethylacetamide (DMA) has been reported to avoid the formation of this by-product during the linker attachment step^69,72^. It should further be noted that performing these reactions at higher temperatures (>120 °C) generally translates into a higher yield of the desired alkylated pomalidomide derivative^71^. Chen et al. similarly determined that the S_N_Ar time can be reduced through its execution in N-methyl-2-pyrrolidone (NMP) under microwave irradiation, but the yields are not better^73^.

To improve yields and chemoselectivity in the production of pomalidomide-based PROTACs, Derksen’s group published an interesting paper investigating the effect of solvent choice on chemical yields^71^. This study demonstrated that the choice of DMSO (polar aprotic solvent) as reaction solvent allows to obtain the best yields with both primary (yield > 50%) and secondary amines (yield > 90%)^71^. Interestingly, it was also found that, in general, with linker bearing the primary amine terminal present lower yields, when compared with the use of secondary amines under the same experimental conditions^71^. This may be due to the formation of the by-product from the displacement of aminoglutarimide and the lower reactivity inherent to primary amines^71^.

There are several examples of cereblon-based PROTACs recently reported in the literature starting from the building block 4-fluorothalidomide (Table 2).

**Table 2:** Examples of pomalidomide-based PROTACs from 3-fluorophtalic anhydride

No.	Chemical structure	Target	Biological activity	Ref.
6		*PD-L1*	*HCC-827 cells* *D*_*max*_ = *61%* In vivo activity *TGI* > 57.35%	^74^
7		*PD-L1*	*HCC-827 cells* *D*_*max*_ = *58%* In vivo activity TGI > *57.35%*	^74^
8		*BRD4*	*MM.1S cells* *IC*_*50*_ = *0.027* *µM* *MV-4-11 cells* *IC*_*50*_ = *0.003* *µM*	^75^
9		GPX4	*MGC803 cells* *D*_*max*_ = 33% *HT-1080 cells* *IC*_*50*_ = 12 µM	^76^
10		SARS-CoV-2 3CL^Pro^	*IC*_*50*_ = 21.2 µM (inhibitory activity)	^77^

Note:

*BRD4* bromodomain-containing protein 4, *GPX4* glutathione peroxidase 4, *PD-L1* programmed death-ligand 1, *SARS-CoV-2 3CL*^*Pro*^ severe acute respiratory syndrome coronavirus 2 3C-like protease, *IC₅₀* half-maximal inhibitory concentration, *DC₅₀* half-maximal degradation concentration, *Dmax* half-maximal achievable degradation, *TGI* tumor growth inhibition concentration.

In 2024, Zang et al. synthesized and evaluated the antitumor activity of novel PROTACs against programmed cell death ligand protein 1 (PD-L1). From the analysis of the structure-activity relationship (SAR) studies, the pomalidomide-based PROTACs showed better results than the lenalidomide-based or VHL-based PROTACs. Among the first, the best PROTACs were those incorporating a polyethylene glycol (PEG) linker with two **(6)** to three units **(7)**, with maximum degradation (D_max_) values of 61% and 58% in HCC-827 cells, respectively. From the results obtained from in vivo studies carried out with the best compounds, a tumor growth inhibition (TGI) up to 57.35% was observed^74^.

In 2023, Zhu et al. published a series of anti-bromodomain-containing protein 4 (BRD4) PROTACs for the treatment of hematological malignancies. The BRD4 protein belongs to the bromodomain-containing (BRD) and extra terminal domain (BET) family, which are known to function as epigenetic readers that regulate the expression of several genes, including tumor-associated genes and anti-apoptotic genes. From the results of the anti-proliferative studies performed, PROTAC **8** was the most potent in MM.1S (half-maximal inhibitory concentration (IC_50_) value of 0.027 µM) and MV-4-11 (IC_50_ = 0.003 µM) cell lines, degrading BRD4 in a time- and concentration-dependent depletion manner. The forced degradation of BRD4 also resulted in a reduction in the expression of the c-MYC protein. Furthermore, it has shown promise in the treatment of pulmonary fibrosis, as it satisfactorily inhibits the activation of MRC5 cells by downregulating the BRD4 protein. It should also be noted that replacing pomalidomide with 4-hydroxylthalidomide reduced the antiproliferative activity in vitro, demonstrating that the chosen cereblon ligand is not indifferent^75^.

In 2023, Qin’s group reported a library of anticancer PROTACs targeting glutathione peroxidase 4 (GPX4). Few GPX4 inhibitors are currently available. However, its inhibition activates cell ferroptosis, a non-apoptotic iron-dependent mechanism of cell death, leading to an effective anticancer effect. Taking advantage of this information, several PROTACs resulting from the binding of ML162 derivatives and ligands to cereblon/VHL E3 ligases were synthesized to evaluate the impact of forced GPX4 degradation on cancer cells. Among them, the cereblon-based PROTAC **9** (GDC-11) (D_max_ = 33%) is the most cytotoxic with an IC_50_ value of 12 µM in HT-1080 cells and is able to induce ferroptosis. VHL-based PROTACs, although having better degradative activity, did not induce any visible cytotoxicity or increased cellular peroxidation of lipids^76^.

A study published by Ciofi-Baffoni’s group reports on a promising cereblon-based peptidomimetic PROTAC targeting the dimeric SARS-CoV-2 3-chymotrypsin-like protease (3CL^Pro^) protein, a key protein in viral replication. By conjugating the GC-376 ligand to a piperazine-piperidine derivative of pomalidomide, they obtained PROTAC **10**, which was able to dramatically degrade the target protein in cultured cells without affecting host cell viability. However, it should be noted that the results obtained with PROTAC are inferior to those observed with inhibitors, such as GC-376 (IC_50_ = 0.19 µM). This study, therefore, highlights the promising therapeutic versatility of the use of cereblon-based PROTACs, not only in the treatment of malignant or neurodegenerative diseases, but also in the treatment of infectious diseases^77^.

#### Pomalidomide-based PROTACs from 3-nitrophthalic anhydride

**Scheme 2:**
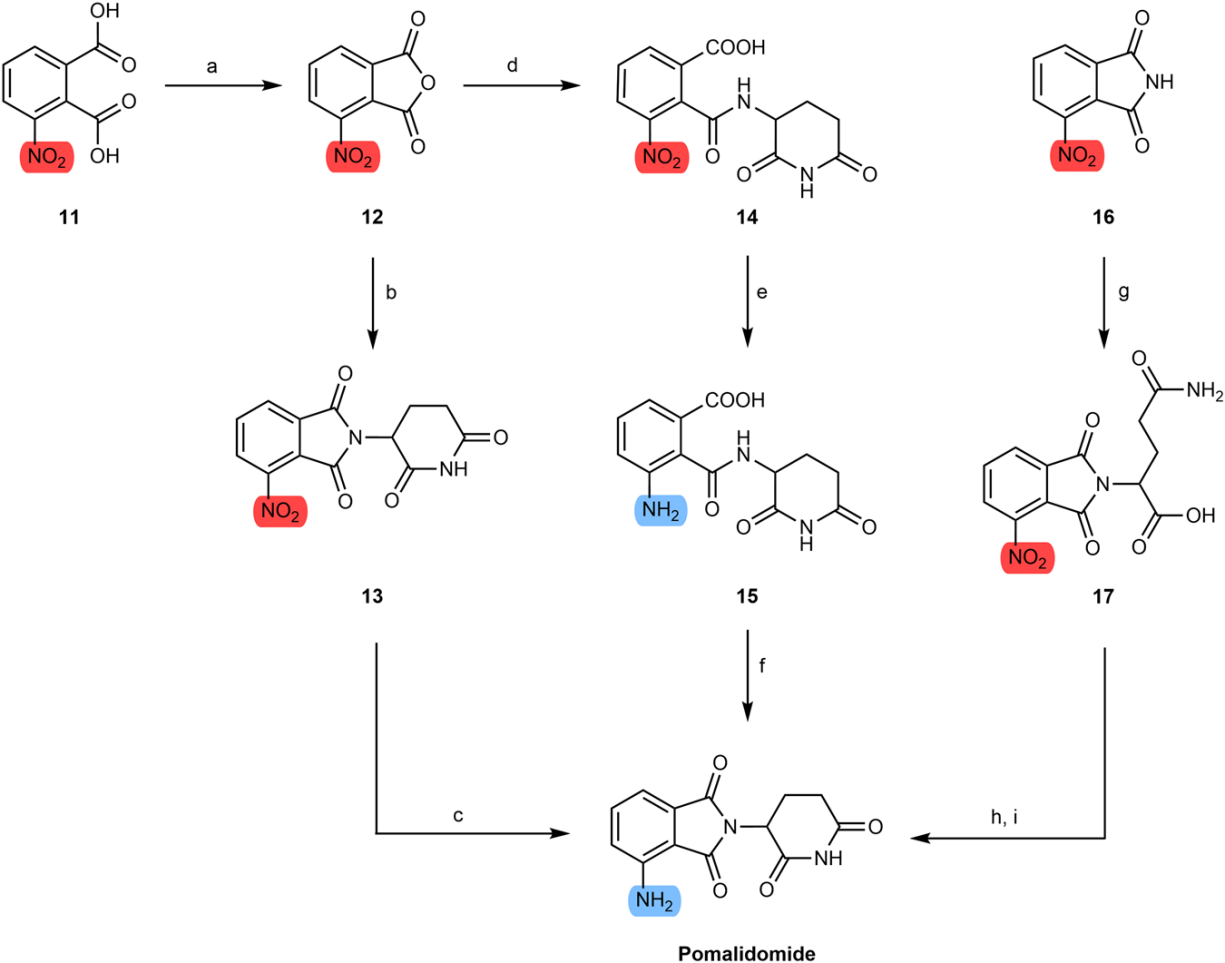
Chemical synthesis of pomalidomide-based PROTACs from 3-nitrophtalic anhydride. Reagents and conditions: **a** Acetic anhydride, reflux, 2 h; **b** tert-butyl-(2,6-dioxopiperidin-3-yl)carbamate **3**, sodium acetate, acetic acid, reflux, 6 h; **b**_**1**_ 3-aminopiperidine-2,6-dionehydrochloride, sodium acetate, acetic acid, 130 °C, 48 h; **b**_**2**_ 3-aminopiperidine-2,6-dionetrifluoroacetate, triethylamine, THF, 80 °C, 6 h; **b**_**3**_ 3-aminopiperidine 2,6-dionehydrochloride, sodium acetate, acetic acid, 80 °C, 12 h; **c**_**1**_ Palladium on Carbon (Pd/C), H_2_, DMF, rt, 24 h; **c**_**2**_ Fe, ammonium chloride, ethanol/water mixture, rt, overnight; **c**_**3**_ Ammonium formate, Pd/C, methanol, rt, 2 h; **d** 3-aminopiperidine-2,6-dionehydrochloride, triethylamine, THF, ≤20 °C, 30 min; **e** Pd/C, H_2_, 145 psi, methanol, rt, 30 min, quant.; **f** Methanol, reflux, 2 h; **g** glutamine, DMF, 80 to 87 °C, 8 h; **h** Pd/C, H_2_, 50 psi, metanol, 3 h; **i** CDI, acetonitrile, reflux, 4.5 h.

As mentioned previously, pomalidomide can be obtained from the condensation between the glutarimide ring and 3-nitrophathalic anhydride **(12)**, either commercially obtained or prepared from 3-nitrophthalic acid **(11)**, thus forming 4-nitrothalidomide **(13)**^78^. The nitro group of 4-nitrothalidomide is then reduced to an amine group, producing pomalidomide, which can then be linked to different types of linkers (Scheme 2)^78^.

There are several methods for the reduction of 4-nitrothalidomide to pomalidomide. Of the various synthetic routes reported, reduction via palladium on carbon (Pd/C)-catalyzed hydrogenation is the one that presents the best yields (around 99%)^79,80^. Other reducing agents may be iron-ammonium chloride or Pd/C and ammonium formate, however, with lower yields^78,81^.

There are another alternative routes for the synthesis of pomalidomide. One such route commences with 3-nitrophtalic anhydride **(12)**, yielding compound 3-aminopiperidine-2,6-dione hydrochloride **(14)**^78^. Subsequent reduction of this compound results in the formation of compound **15**, which undergoes cyclization to yield the desired product^78^. This method has been observed to produce pomalidomide with good yields (>60%)^78^. An alternative approach involves the initiation of the reaction with 3-nitrophthalimide **(16)**, which, upon reacting with glutamine via amination, results in the formation of compound **17**^78^. This compound undergoes reduction and cyclization, leading to the production of pomalidomide, albeit with a low yield due to the poor chemical reduction^78^.

Steinebach and colleagues published a series of cereblon homo-PROTACs, i.e., a cereblon-based PROTAC targeting cereblon itself. Using 4-nitrothalidomide as the starting building block, PROTACs were synthesized comprising two pomalidomide moieties linked by different types of linker. The homodimerized PROTAC **18** was characterized as a potent and long-lasting degrader of cereblon protein at low concentrations by western blot studies (Table 3)^80^.

**Table 3:** Examples of pomalidomide-based PROTACs from 3-nitrophthalic anhydride

No.	Chemical structure	Target	Biological activity	Ref.
18		Cereblon	*MM.1S and NCI-H929 cells* Degradation at nanomolar concentration	^80^
19		CK2	*MDA-MB-231 cells* *D*_*max*_ > 80% *IC*_*50*_ = 17.36 µM Inhibition rate = 88.2% (enzymatic activity)	^81^

Note:

*CK2* casein kinase 2, *IC*_*50*_ half-maximal inhibitory concentration, *D*_*max*_ half-maximal achievable degradation.

Another example of PROTACs starting from 3-nitrophthalic anhydride are the compounds published by the Gou group. Their paper reported on PROTACs targeting anti-casein kinase 2 (CK2), the serine/threonine protein kinase associated with tumor growth that is overexpressed in cancer cells. The reaction of pomalidomide with chloroacetyl chloride and then with sodium azide is followed by click-chemistry reaction to give triazole-based PROTACs. Among these, PROTAC **19** shows significant UPS-dependent degradation of the CK2 protein. By degrading the target, PROTAC **19** reduces AKT protein phosphorylation and leads to increased p53 expression^81^.

#### N-alkyl and acyl-connected pomalidomide-based PROTACs

**Scheme 3:**
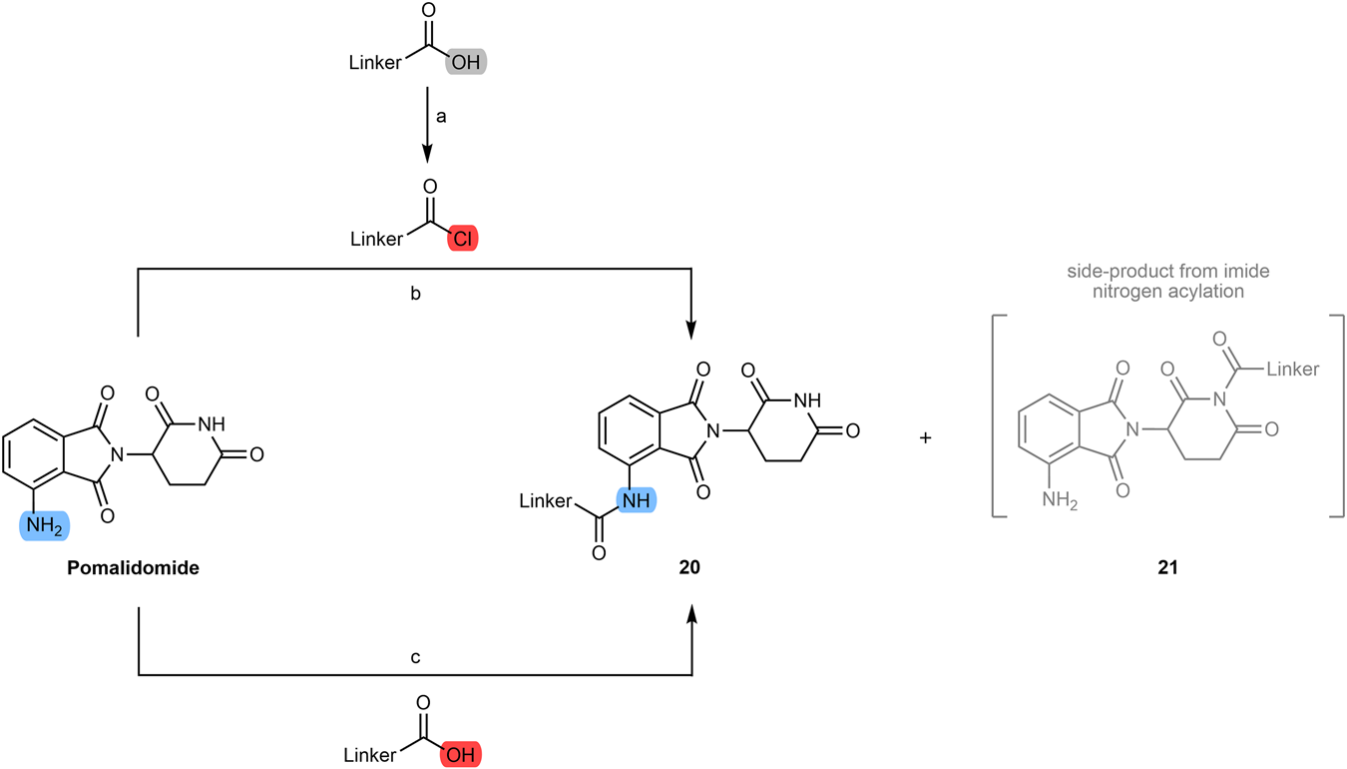
Chemical synthesis of N-alkyl and acyl-connected pomalidomide-based PROTACs. Reagents and conditions: **a** Thionyl chloride, DMF or THF, reflux, 4 h; **b** THF, reflux, various times depending on the type of linker (4 h to overnight); **c**_**1**_ HOBT, EDC, triethylamine, THF or DMF, rt, 24 h (time varies depending on the type of linker); **c**_**2**_ HATU, DIPEA, DMF, rt, 24 h (time varies depending on the type of linker).

When the cereblon ligand to be incorporated into PROTAC is pomalidomide, the most commonly reported synthetic route for coupling to the linker is via acylation reactions with activated acyl donors. Attaching the aniline group of pomalidomide to an acyl chloride-bearing linker generated in situ in THF under reflux is undoubtedly the most common route (Scheme 3)^79,82,83^. Note also that there is the possibility to react carboxylic acid linkers directly with pomalidomide using coupling agents such as 1-hydroxybenzotriazole (HOBt), 1-ethyl-3-(3-dimethylaminopropyl)carbodiimide (EDC), hexafluorophosphate azabenzotriazole tetramethyl uronium (HATU), 1-hydroxy-7-azabenzotriazole (HOAt), among others. However, it is necessary to consider the possibility of the formation of the secondary product **21** resulting from the acylation of the imide nitrogen, which reduces the yield of the desired product.

**Scheme 4:**
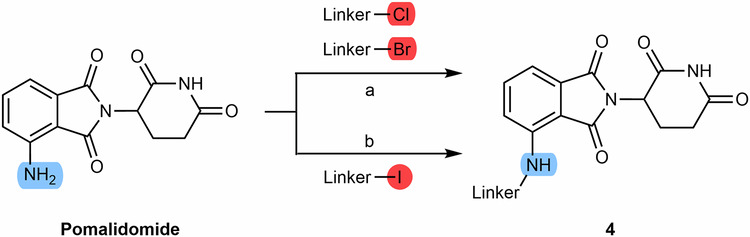
Chemical synthesis of N-alkyl connected pomalidomide-based PROTACs starting with pomalidomide. Reagents and conditions: **a** DIPEA, N-methyl-2-pyrrolidone (NMP), 110 °C, 6 h; **b** DIPEA, DMF, rt, 16 h.

Besides the acylation of pomalidomide, another possibility is to perform linker attachment by N-alkylating the amine group of pomalidomide using alkylhalide linkers (Scheme 4)^84^. Unfortunately, this strategy is not only poorly chemoselective, but also has low chemical yields due to low nucleophilicity^71^.

In 2023, Wu et al. reported a series of cereblon-based PROTACs targeting heat shock protein 90 isoform α (HSP90α) for the treatment of breast cancer. HSP90 is a widely expressed molecular chaperone protein responsible for maintaining the correct folding of various proteins and is therefore associated with the stability and activation of oncoproteins. It is also known that the α-isoform of HSP90 is overexpressed in invasive tumors and is associated with metastasis. Given its role in tumor development and the fact that most HSP90 inhibitors are highly toxic, the therapeutic potential of degradation via PROTACs was investigated. In vitro studies have shown that PROTAC **22**, which results from the attachment of pomalidomide via an alkyl linker to the AT13387 inhibitor derivative, selectively degrades HSP90α compared with other isoforms, with a degradation potency in the micromolar range (half-maximal target degradation (DC_50_) value of 0.295 μM, D_max_ = 66.4%). In vivo studies showed a tumor inhibition rate of 53.99%, higher than the parent inhibitor AT13387 (43%), with reduced toxicity. Pharmacokinetic (PK) assays in rats showed that PROTAC **22** has a plasma half-life of 150 min and a maximum plasma drug concentration (C_max_) of 302 ng/mL after a 15 mg/kg dose (Table 4)^85^.

**Table 4:** Examples of N-alkyl and acyl-connected pomalidomide-based PROTACs

No.	Chemical structure	Target	Biological activity	Ref.
22		HSP90α	*MCF-7 cells* *DC*_*50*_ = 0.295 μM *D*_*max*_ = 66.4% In vivo activity TGI = 53.99%	^85^
23		ERK1/2	*HCT116 cells* *DC*_*50*_ = 102 nM *IC*_*50*_ = 2.2 µM In vivo activity	^86^
24		PTP1B	*HepG2 cells* *DC*_*50*_ (48 h) = 0.20 µM *DC*_*50*_ (72 h) = 0.05 In vivo activity	^87^
25		CDK4/6	*Jurkat cells* *DC*_*50*_ (CDK6) = 2.5 nM *D*_*max*_ (CDK6) = 96% *DC*_*50*_ (CDK4) = 10.5 nM *D*_*max*_ (CDK4) = 100% *IC*_*50*_ = 0.18 µM	^88^
26		DYRK1	*HEK293 cells* *DC*_50_ (DYRK1A) = 12.3 nM *D*_*max*_ (DYRK1A) ∼ 90% *D*_*max*_ (DYRK1B) ∼ 50%	^89^
27		ALK	*Karpas299 cells* *DC*_*50*_ (NPM-ALK) = 4.6 nM *D*_*max*_ (NPM-ALK) = 92% *H3122 cells* *DC*_*50*_ (EML4-ALK) = 357.6 nM *D*_*max*_ (NPM-ALK) = 81.34% In vivo activity	^91^
28		BCL-6	*SU-DHL-4 cells* *DC*_*50*_ = 676.1 nM *D*_*max*_ = 93.0% *IC*_*50*_ = 399.0 nM *DOHH2 cells* *DC*_*50*_ = 557.7 nM *D*_*max*_ = 93.7% *IC*_*50*_ = 56.2 nM In vivo activity TGI = 71.8%	^228^
29		CBP	*MV4-11 cells* *DC*_*50*_ < 1.9 nM *IC*_*50*_ = 0.9 nM In vivo activity TGI = 93%	^229^
30		eIF4E	*Cells stably expressing the BRD4*_*BD2*_*-GFP with mCherry reporter* *IC*_*50*_ = 1.57 µM (Cereblon protein)	^134^
31		HDAC1	*HCT116 cells* *D*_*max*_ < 50%	^230^
32		KRAS	*H358 cells* *IC*_*50*_ = 0.51 µM *MiaPaca-2* *IC*_*50*_ = 4.14 µM *MiaPaca-2 (GILA)* *IC*_*50*_ = 0.51 µM	^231^

Note:

*ALK* anaplastic lymphoma kinase, *BCL-6* B-cell lymphoma 6, *CBP* CREB-binding protein, *CDK4/6* cyclin-dependent kinase 4/6, *DYRK1* dual-specificity tyrosine-regulated kinase 1, *eIF4E* eukaryotic translation initiation factor 4E, *ERK1/2* extracellular signal-regulated kinase 1/2, *GILA* growth in low attachment, *HDAC1* histone acetylation and deacetylation 1, *HSP90α* heat shock protein 90 alpha, *KRAS* kristen rat sarcoma virus, *PTP1B* protein tyrosine phosphatase 1B, *IC₅₀* half-maximal inhibitory concentration, *DC₅₀* half-maximal degradation concentration, *Dmax* half-maximal achievable degradation, *TGI* tumor growth inhibition concentration.

Meng’s group (2023) investigated the ability of PROTACs to degrade extracellular signal-regulated protein kinases 1/2 (ERK1/2). Since these proteins regulate the growth, proliferation and differentiation of cells, their degradation is seen as a way to combat cancer cells. Of the various PROTACs obtained, which recruit different E3 ligases, the cereblon-based PROTACs showed the best degradation activity. The best compound was the pomalidomide-based PROTAC **23**, which was able to degrade targets with nanomolar potency (DC_50_ value of 102 nM) and inhibit cell proliferation (IC_50_ value of 2.2 µM) of HCT116 cancer cells. PROTAC **23** also demonstrated the ability to inhibit tumor cell migration. In vivo xenograft studies showed significant tumor regression after treatment with this degrader, exceeding the results obtained with 5-fluorouracil^86^.

In 2024, Fang et al. discovered a selective long-term hypoglycemic pomalidomide-based PROTAC targeting protein tyrosine phosphatase 1B (PTP1B), with enormous potential for the treatment of PTP1B-mediated insulin sensitivity. The PTP1B protein is a non-receptor protein tyrosine phosphatase that plays a negative regulatory role in several intracellular signaling pathways, including insulin and leptin signaling. By inhibiting the phosphatase action of PTP1B on the insulin receptor (IR) and insulin receptor substrates (IRS) proteins, insulin sensitivity is increased, and consequently better glycemic control is generated. Unfortunately, the discovery of inhibitors for this promising therapeutic target for diabetes has been hampered by problems of bioavailability and selectivity. The authors therefore investigated the biological activity of a library of different anti-PTP1B degraders obtained by acylating pomalidomide via the acid chloride linker, followed by linkage via an amide to different PT1B inhibitors. Western blot analysis showed significant target degradation induced by the action of PROTACs in HepG2 cells. PROTAC **24** with the longer PEG-based linker (23 atoms) showed remarkable UPS-dependent degradation potency with DC_50_ values of 0.20 µM (48 h) and 0.05 (72 h). Additional studies in HepG2 cells further demonstrated that PROTAC **24** is selective and induces long-term hypoglycemic activity via activation of the IRS-1/PI3K/Akt signaling pathway. In addition, oral glucose tolerance test results in mice showed a significant reduction in blood glucose (29%)^87^.

In 2024, Chen and co-workers developed cereblon-based PROTACs that target the degradation of cyclin-dependent kinases 4 and 6 (CDK4/6). The CDK6 protein is often involved in controlling the G1/S transition of the cell cycle and is overexpressed and hyperactivated in several types of cancer, ultimately promoting uncontrolled cell growth of cancer cells. Several ATP-competitive CDK6 inhibitors (e.g., palbociclib, ribociclib) have been reported, but these are not very selective and are associated with serious side effects, such as neutropenia. Furthermore, the emergence of resistance is one of the shortcomings associated with the use of inhibitors. Different degraders have been reported resulting from the binding of the CDK6 ligand YY173 to pomalidomide with different types of linkers, via N-alkylation or acylation of the aniline group. In vitro degradation studies have shown that PROTAC **25** is able to rapidly induce the degradation of CDK6 (DC_50_ = 2.5 nM, D_max_ = 96%) and CDK4 (DC_50_ = 10.5 nM, D_max_ = 100%) proteins. By promoting the degradation of the target protein, PROTAC **25** inhibits cell proliferation of jurkat cells (IC_50_ value of 0.18 µM), induces apoptosis and causes cell cycle arrest in the G1 phase in a dose-dependent manner. Notably, the cell inhibition results exceed those obtained with the isolated parent inhibitor (IC_50_ = 1.47 µM) or palbociclib (IC_50_ = 1.50 µM)^88^.

Taking advantage of the benefits presented by induced protein degradation, Hulme et al. designed and synthesized a first-in-class dual-specificity tyrosine phosphorylation-regulated kinase (DYRK1) PROTAC. DYRK1 is a protein kinase involved in signaling pathways that promote cell proliferation and differentiation, so its deregulation is associated with the promotion of tumor growth. Overactivation of this protein is also associated with the development of neurodegenerative diseases, such as Alzheimer’s disease, as well as cognitive deficit. DYRK1A has also been identified as a target of interest for the treatment of inflammatory, metabolic, cardiovascular and infectious diseases. In this paper, the authors linked the ATP-competitive inhibitor DYRK1 to pomalidomide or a VHL ligand via various PEG-based linkers. Cereblon-based PROTAC **26** (DYR684) induced rapid, potent and selective UPS-dependent degradation of the DYRK1 protein, resulting in effective suppression of the DYRK1 pathway, superior to that observed with classical type 1 DYRK1 inhibitors. In addition, the lead compound DYR684 is metabolically stable and has a pharmacokinetic profile suitable for in vivo application^89^.

#### Introduction of vectors to facilitate the construction of PROTACs

**Scheme 5:**
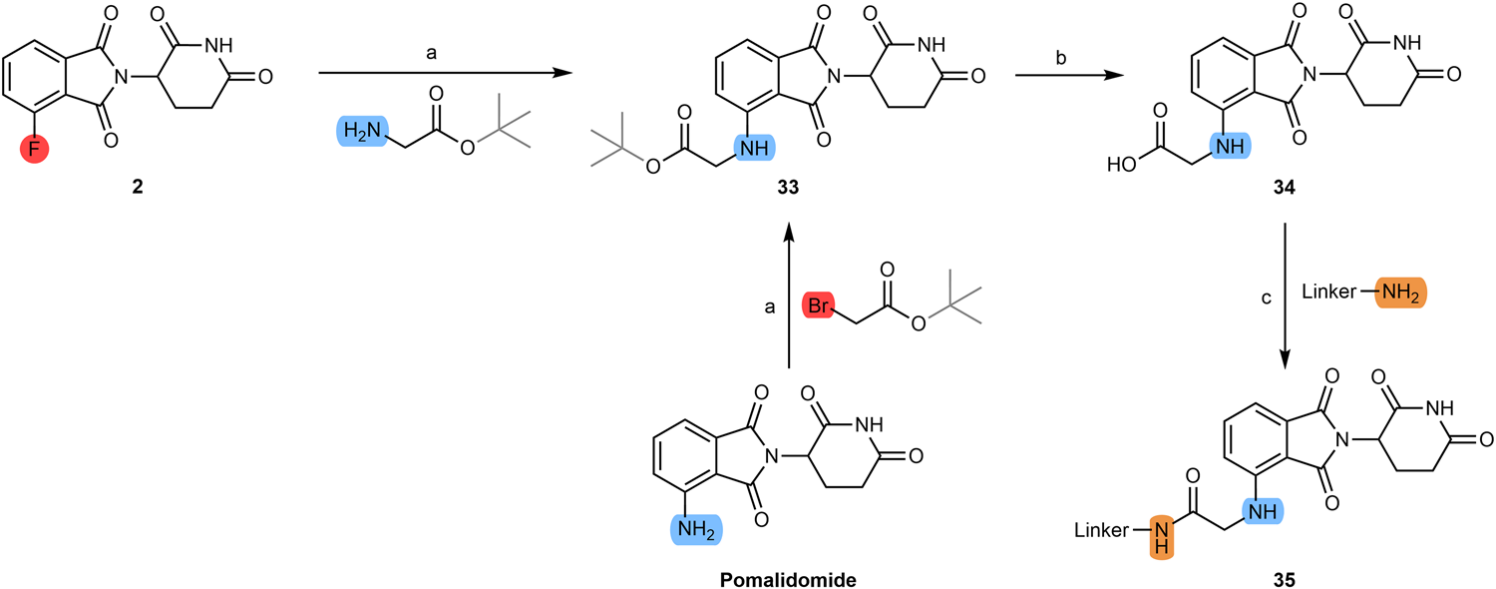
Chemical synthesis of pomalidomide-based PROTACs with two-carbon spacer to facilitate linker attachment. Reagents and conditions: **a** DIPEA, DMF, 90 °C, 12 h; **b** Dichloromethane/trifluoroacetic acid (1:1), rt, 2 h; **c**_**1**_ i. Thionyl chloride, DMF or THF, reflux, 4 h; ii. THF, reflux, various times depending on the type of linker (4 h to overnight); **c**_**2**_ HOBT, EDC, triethylamine, THF or DMF, rt, 24 h (time varies depending on the type of linker); **c**_**3**_ HATU, DIPEA, DMF, rt, 24 h (time varies depending on the type of linker).

Another possible strategy is to introduce groups that act as vectors to facilitate the attachment of the cereblon ligand to the linker. For example, the reaction between 4-fluorothalidomide **(2)** and tert-butyl-2-aminoacetate or the reaction between pomalidomide and tert-butylbromoacetate results in the introduction of an acetamide group **(33)**, which facilitates the attachment of a linker with an amine terminus via the formation of an amide bond **(35)** (Scheme 5)^68,90^.

For example, Gong and colleagues designed and synthesized a novel anti-anaplastic lymphoma kinase (ALK) degrader, PROTAC **27**, in which the piperazine moiety of the target ligand was directly linked to the acetamide group of (2-(2,6-dioxopiperidin-3-yl)-1,3-dioxoisoindolin-4-yl)glycine using the coupling agent HATU in DIPEA and DMF overnight. Although several small molecule inhibitors of ALK are approved with excellent therapeutic efficacy, the emergence of resistance-conferring mutations limits their clinical use. PROTACs could therefore be an alternative. In vitro studies with PROTAC **27** have shown that it is able to degrade three types of ALK mutants (NPM-ALK, EML4-ALK and F1174L mutations). In addition to its cytotoxicity and selectivity for ALK-positive cells, PROTAC **27** demonstrated potent anti-cancer activity in a xenograft mouse model, superior to that achieved with the inhibitor ceritinib^91^. From this study, it can be concluded that the degradation of a POI, induced by PROTAC’s forced approach to an E3 ligase, could be a promising alternative strategy to the loss of clinical efficacy of inhibitors due to the development of resistance.

**Scheme 6:**
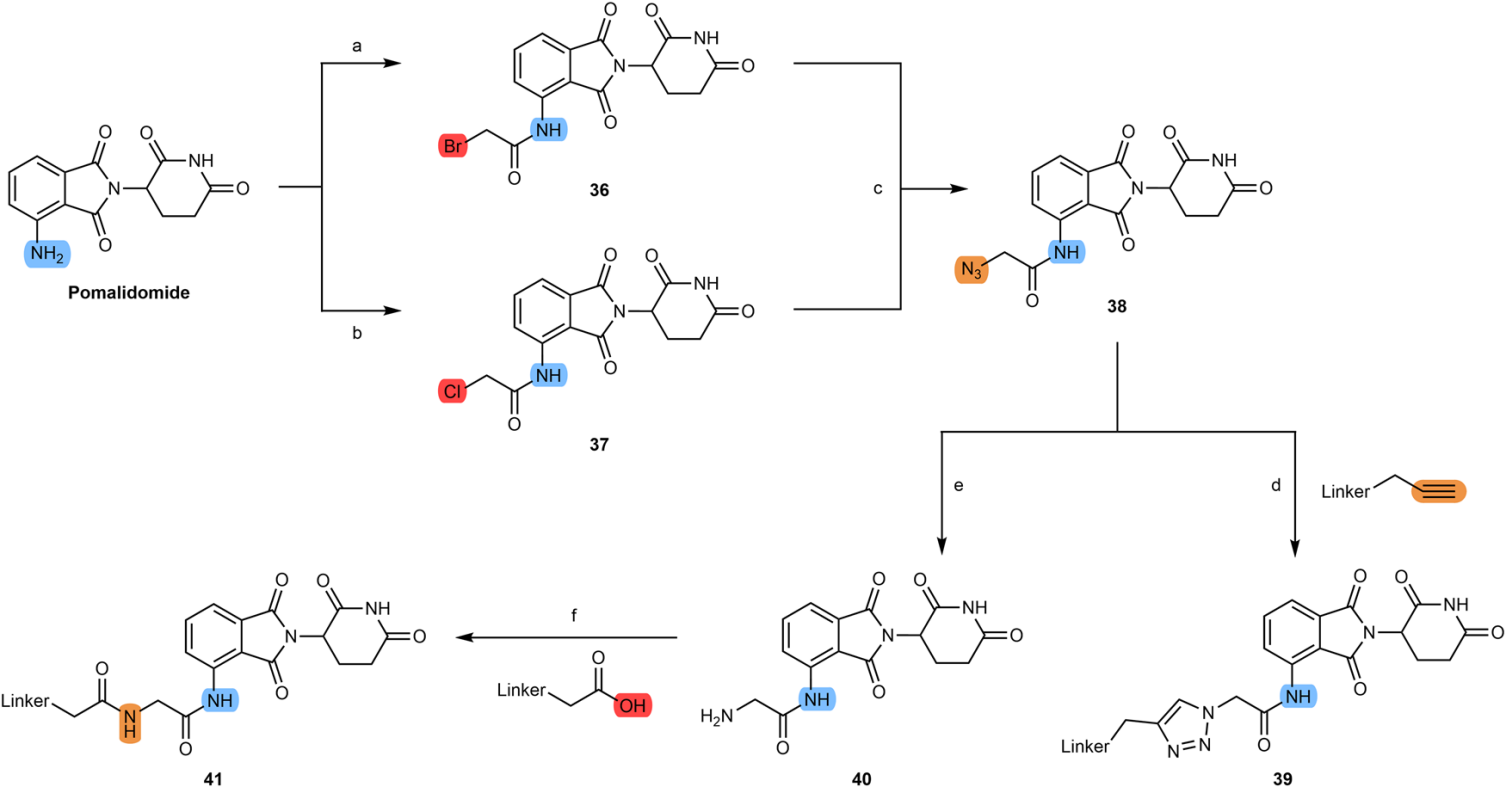
Chemical synthesis of pomalidomide derivatives for acyl-connected based PROTACs and click-chemistry PROTACs. Reagents and conditions: **a** bromoacetyl chloride, THF, reflux, overnight; **b** chloroacetyl chloride, THF, reflux, overnight; **c** Sodium azide, acetone, reflux, overnight; **d** sodium ascorbate, Copper (II) sulfate × 5 H_2_O, THF/H_2_O, rt, overnight (depending on the type of linker); **e** Palladium on Carbon (Pd/C), H_2_, methanol, rt, 6 h; **f** TBTU, triethylamine, DMF, 50 °C, 24 h (depending on the type of linker).

Another possibility is to react pomalidomide with haloacetyl halides such as bromoacetyl chloride or chloroacetyl chloride to give compounds **36** and **37**. Compounds **36** and **37** can be used for example to alkylate amine-bearing linkers. Another possible route is to form azide **38** by refluxing compounds **36** and **37** with sodium azide in acetone overnight. Compound **38** can be used as a building block for the construction of click-chemistry based PROTACs through its reaction with an alkyne terminal linker **(39)**. Compound **38** can also be reduced to amine **40**, which facilitates the attachment of the linker to the carboxylic acid terminal via the formation of an amide bond **(41)** (Scheme 6)^81,83^.

Although amide bonds are often used to link the various components of PROTACs, it should be noted that these bonds are still susceptible to hydrolytic or enzymatic cleavage, resulting in a loss of potency of the PROTAC^92^. In addition, this approach adds additional polar surface area that can create physicochemical properties that may hinder cell penetration^92–94^.

### Thalidomide-based PROTACs

Thalidomide-based PROTACs can be obtained by incorporating 5-aminothalidomide derivatives derived from the alkylation of 5-fluorothalidomide^95^. Another strategy is to use 4-hydroxythalidomide as the starting building block to create ether-linked 4-hydroxythalidomide derivatives^96^.

#### 5-Aminothalidomide based PROTACs

**Scheme 7:**
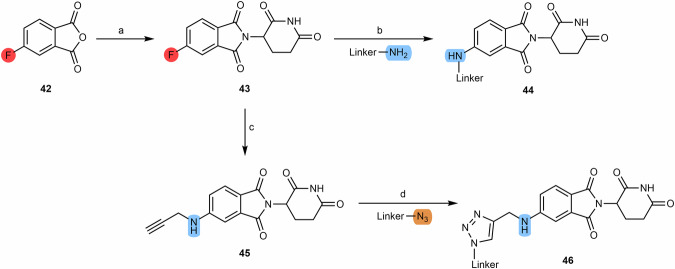
Chemical synthesis of 5-Aminothalidomide based PROTACs. Reagents and conditions: **a** 3-aminopiperidine-2,6-dionehydrochloride, potassium acetate, acetic acid, 90 °C, overnight; **b** DIPEA, N-methyl-2-pyrrolidone (NMP), 90 °C, overnight (depending on the type of linker); **c** Propargylamine, DIPEA, DMF, 90 °C, 12 h; **d** Copper (II) sulfate × 5 H_2_O, sodium ascorbate, H_2_O/tert-butanol, rt, 16 h (depending on the type of linker).

Although less commonly reported in cereblon-based PROTACs, 5-aminothalidomide derivatives are compounds that also have the ability to bind to cereblon E3 ligase. The synthesis of 5-aminothalidomide derivatives from the substrate 5-fluorothalidomide **(43)** is very similar to the synthetic route from the 4-fluoro analog, both in terms of reaction conditions and reagents^97^. Thus, from the S_N_Ar between 5-fluorothalidomide **(43)** and primary amines linkers, in the presence of DIPEA and under heating, it is possible to obtain several 5-aminothalidomide derivatives **(44)** (Scheme 7)^96,98,99^. However, the yields are slightly lower than those obtained with the 4-fluoro analog due to the lower electron-withdrawing effect of the carbonyl groups in this position. It is also reported that from the reaction between 5,6-difluorothalidomide with an equimolar amount of nucleophile it is possible to obtain mono-substituted derivatives^100^. From the reaction between 5-fluorothalidomide **(43)** and propargylamine, it is possible to obtain compound **45**, which can be used as a building block for the construction of click-chemistry based PROTACs.

**Scheme 8:**
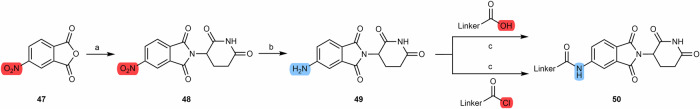
Chemical synthesis of acyl-connected 5-Aminothalidomide based PROTACs. Reagents and conditions: **a** 3-aminopiperidine-2,6-dione trifluoroacetate, acetic acid, reflux, 2 h; **b** Palladium on Carbon (Pd/C), H_2_, rt, 20 h; **c** THF, reflux, 4 h (depending on the type of linker).

From the condensation of 4-nitrophthalic anhydride **(47)** with 3-aminopiperidine-2,6-dione trifluoroacetate, followed by reduction of the product formed **(48)**, it is possible to obtain the 5-aminothalidomide derivative **(49)** by an alternative route (Scheme 8)^101^. As previously described for pomalidomide, new derivatives can be synthesized from 5-aminothalidomide **(49)** by acylating the primary amine with acyl chloride linkers, or directly with a carboxylic acid terminal linker, leading to compound **50**. Another option is to alkylate the amine group of 5-aminothalidomide with alkylhalide linkers^102^. However, similar to the synthesis of pomalidomide derivatives, there are still problems related to the lack of chemoselectivity.

In 2024, three different groups published several examples of 5-aminothalidomide-based PROTACs targeting Fms-like protein tyrosine receptor kinase 3 (FLT3), an established target in acute myeloid leukemia (AML)^103^. The tyrosine kinase receptor FLT3 gene is frequently mutated in AML patients (20–30%), with internal tandem duplication (ITD) being the most common FLT3 mutation (25% of AML cases)^104,105^. Many of the mutations result in constitutive activation of FLT3, promoting proliferation, differentiation and survival of leukemic cells^105^. Several FLT3 protein inhibitors are approved for the treatment of AML (e.g., quizartinib, gilterinib), but loss of clinical efficacy due to the emergence of conferring resistance mutations is common, resulting in high leukemic burden and poor prognosis^105^.

Hu’s group reported the orally bioavailable anti-AML PROTAC **51**, capable of inducing potent and selective UPS-dependent degradation of FLT3-ITD protein (DC_50_ of 7.4 nM in MV4-11 cells), with a long-lasting and dose-dependent effect in vitro and in vivo. In vitro studies showed that PROTAC **51** has potent cytotoxic activity against MV4-11 (IC_50_ = 39.9 nM) and MOLM-13 (IC_50_ = 169.9 nM) cells, causing cell cycle delay in the G1 phase and inducing apoptosis. With a desirable PK profile (e.g., half-life time = 3.8 h) combined with good oral bioavailability (53%), PROTAC **51** induced dramatic tumor regression in subcutaneous AML xenograft models. In vivo studies show that PROTAC **51** eliminates CD45^+^ CD33^+^ human leukemic cells and prolongs the life of the mice tested. It is also worth noting that in drug-resistant AML cells, the anti-proliferative activity of PROTAC **51** is superior to that obtained with FLT3 inhibitors (Table 5)^106^.

**Table 5:** Examples of 5-Aminothalidomide based PROTACs

No.	Chemical structure	Target	Biological activity	Ref.
51		FLT3-ITD	*MV4-11 cells* *DC*_*50*_ = 7.4 nm *IC*_*50*_ = 39.9 nM *MOLM-13* *IC*_*50*_ = 169.9 nM In vivo activity	^106^
52		FLT3-ITD	*FLT3-ITD mutant AML cells* *DC*_*50*_ = 41.35 nM *IC*_*50*_ = 1.66 nM In vivo activity TGI = 77%	^107^
53		FLT3 CDK2	*MV4-11 cells* *DC*_50_ (FLT3) = 5.69 nM *D*_*max*_ (FLT3-ITD) = 77% *IC*_*50*_ = 2.90 nM *HL-60 cells* *DC*_*50*_ (CDK2) = 12.6 nM *D*_*max*_ (CDK2) = 82% *THP-1 cells* *IC*_*50*_ = 37.08 nM	^108^
54		AR	*Prostate cancer cells* *DC*_*50*_ = 2.4 nM *D*_*max*_ = 96%	^109^
55		GSPT1	*MOLM13 cells* *DC*_*50*_ = 5.25 nM *IC*_*50*_ = 8.55 nM *NB4 cells* *IC*_*50*_ = 2.69 nM *HL-60 cells* *IC*_*50*_ = 8.19 nM *SU-DHL-1 cells* *IC*_*50*_ = 6.29 nM In vivo activity	^232^

Note:

*AR* androgen receptor, *FLT3* Fms-like tyrosine kinase 3, *FLT3-ITD* Fms-like tyrosine kinase 3 internal tandem duplication, *GSPT1* G1 to S phase transition 1, *IC₅₀* half-maximal inhibitory concentration, *DC₅₀* half-maximal degradation concentration, *Dmax* half-maximal achievable degradation, *TGI* tumor growth inhibition concentration.

From the analysis of the SAR studies of the reported FLT3 degraders, it is possible to verify that the cereblon-based PROTAC presented better results than the VHL-based counterparts. Within the cereblon-based PROTACs, the best ones were those with azacyclic linkers (such as piperazine, piperidine, pyrrolidine, and azetidine) instead of flexible linkers^106^.

In that same year, Hu et al. published another anti-FLT3 PROTAC **52**, which results from the binding of the inhibitor gilterinib with 5-fluorothalidomide via 4-methylpiperidine linker. Similar to PROTAC **51**, PROTAC **52** exhibits robust cytotoxic activity against several FLT3-ITD mutant AML cells (IC_50_ = 1.66 nM) by inducing a marked UPS-dependent degradation of the FLT3-ITD protein (DC_50_ = 41.35 nM). PROTAC **52**, by forcing the degradation of the FLT3-ITD protein, blocked the cell cycle in the G1 phase and induced apoptosis. Oral administration of PROTAC **52** in MV-4-11 xenograft models produced a good antitumor effect with tumor growth inhibition of around 77%, although lower than that obtained with the isolated use of the parent warhead gilterinib due to the poor pharmacokinetic characteristics of PROTAC (oral bioavailability of 5%). As previously observed, also in this study the use of rigid azacyclic linkers resulted in better degradation activities. Furthermore, the attachment site of the linker on thalidomide (4′ or 5′ position) is not indifferent, in which case binding to the 4′ position of thalidomide is favored. It is important to highlight that both PROTACs **51** and **52** demonstrate that PROTACs are a promising therapeutic alternative capable of overcoming the problem of drug resistance^107^.

Dong’s group reported also a potent thalidomide-based PROTAC **53** with a 4-methylpiperidine linker, corroborating that the use of azacyclic linkers favors the degradational activity of FLT3 degraders. PROTAC **53** induced the degradation of FLT3 protein (DC_50_ = 5.69 nM), including FLT3-ITD (D_max_ = 77%), as well as CDK2/4/6/9 in MV4-11 cells. Of note is the degradation of CDK2 in HL-60 cells with DC_50_ values of 12.6 nM and D_max_ of 82%. In addition, PROTAC **53** demonstrated robust cytotoxicity against a panel of AML cell lines with IC_50_ values ranging from 2.90 nM (MV4-11 cells) to 37.08 nM (THP-1 cells). In vivo studies with this PROTAC have not been presented^108^.

In 2024, a 5-aminothalidomide based PROTACs against the androgen receptor (AR) designated as UBX-390 **(54)** was published by Lee’s group. In vitro studies in prostate cancer cells with PROTAC UBX-390 (DC_50_ = 2.4 nM, D_max_ = 96%) demonstrated that it has a prolonged degradation activity superior to that obtained with predecessor PROTACs, such as **ARV-110** (DC_50_ = 3.1 nM, D_max_ = 96%) which is currently in clinical studies. Furthermore, PROTAC UBX-390 remains active when tested in resistant prostate cancer cells, thus showing potential for the treatment of castration-resistant prostate cancer^109^.

#### 4-Hydroxythalidomide based PROTACs

By exploiting the reactivity of the phenolic group of 4-hydroxythalidomide, its direct alkylation allows the synthesis of diverse and versatile starting building blocks for subsequent incorporation into the construction of cereblon-based PROTACs, e.g., via amidation, alkylation or click-chemistry reactions^97,110–112^.

##### Eter-connected thalidomide-based PROTACs

**Scheme 9:**
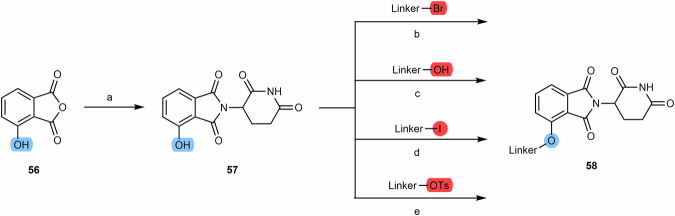
Chemical synthesis of eter-connected thalidomide-based PROTACs. Reagents and conditions: **a**_**1**_ 3-aminopiperidine-2,6-dionehydrochloride, potassium acetate, acetic acid, reflux, 24 h; **a**_**2**_ 3-aminopiperidine-2,6-dionehydrochloride, triethylamine, toluene, reflux, 12 h; **a**_**3**_ i. 3-aminopiperidine-2,6-dione, triethylamine, DMF, reflux, 4 h; ii. DCC, reflux, 72 h; **b** Sodium bicarbonate, Potassium Iodide, DMF, 60 °C, 12 h; **c** Triphenylphosphine (PPh_3_), di-tert-butyl azodicarboxylate (DBAD), THF, rt, 2 h; **d** Sodium bicarbonate, DMF, 70 °C, 6 h; **e** DMF, 80 °C, 16 h. (Note: conditions may vary depending on the type of linker).

The synthesis of 4-hydroxythalidomide **(57)** can be obtained by condensation between 3-hydroxyphthalo anhydride **(56)** and the glutarimide ring, under different conditions and reagents (Scheme 9)^110–112^. After obtaining 4-hydroxythalidomide, it is possible to form ether bond-containing derivatives through its reaction with a linker whose terminus is iodine, bromine or tosylate group (compound **58**)^79,113–115^. Since the phenolic group of 4-hydroxythalidomide can act as a nucleophile in the presence of reagents such as triphenylphosphine (TPP or PPh_3_) and diisopropylazodicarboxylate (DIAD) or similar, it is possible to introduce linkers bearing a primary or secondary alcohols, via Mitsunobu reactions^93,116^. However, it should be taken into account that although the difference in the pKa values of the two acidic groups of 4-hydroxythalidomide is sufficient to ensure selective alkylation of the phenolic group, a reaction may still occur in the imide nitrogen zone given its adequately acidity. To avoid side reactions with imide nitrogen, it is crucial to choose an appropriate base (e.g., sodium bicarbonate, sodium carbonate)^117^. Another possible strategy is to introduce a *tert*-butyloxycarbonyl (Boc)-protecting group at this position^113^.

Several Cereblon-based PROTACs containing ether-linked 4-hydroxythalidomide derivatives have been reported in scientific literature (Table 6).

**Table 6:** Examples of 4-Hydroxythalidomide based PROTACs

No.	Chemical structure	Target	Biological activity	Ref.
59		*EGFR*^*DEL19*^	*Hypoxia* *IC*_*50*_ = 1.09 µM *Normoxia* *IC*_*50*_ = 1.92 µM	^118^
60		*EGFR*^*DEL19*^	*Hypoxia* *IC*_*50*_ = 0.61 µM *Normoxia* *IC*_*50*_ = 1.10 µM	^118^
61		FLT3	*MV4-11 cells* *DC*_*50*_ = 0.64 nM *D*_*max*_ = 95% *IC*_*50*_ = 1.50 nM In vivo activity	^119^
62		20S proteasome subunit β5	*FaDu cells* *DC*_*50*_ = 0.11 µM *D*_*max*_ = 87% In vivo activity	^124^
63		20S proteasome subunit β5	*FaDu cells* *DC*_*50*_ = 0.16 µM *D*_*max*_ = 85% In vivo activity	^124^
64		Aurora-A	*MV4-11 cells* *DC*_*50*_ = 28 nM *DC*_*max*_ = 300 nM	^55^

Note:

*EGFR*^*DEL19*^ Epidermal growth factor receptor exon 19 deletion, *FLT3* Fms-like tyrosine kinase 3, *IC₅₀* half-maximal inhibitory concentration, *DC₅₀* half-maximal degradation concentration, *DC*_*max*_ maximal degradation concentration, *Dmax* half-maximal achievable degradation.

In 2023, Zhang’s group reported two innovative tumor hypoxia-activated PROTACs **59** and **60** targeting epidermal growth factor receptor 19 deletions (EGFR^DEL19^). Due to the abnormal vasculature of solid tumors, they present an environment with low oxygen levels (hypoxic environment), which makes it difficult to deliver drugs to the site of action and promotes metastasis of cancer cells. Thus, the present group incorporated hypoxia-activated leaving groups (HALGs), such as (1-methyl-2-nitro-1H-imidazol-5-yl)methyl or 4-nitrobenzyl groups, into ether-linked thalidomide-based PROTACs. Because HALGs were added to the nitrogen of the imide group, these PROTACs are inactive under normoxic conditions and are unable to recruit the cereblon protein. Under hypoxic conditions, the HALGs are cleaved and the PROTAC becomes active, degrading the EGFR^DEL19^ protein. In vitro studies have shown that PROTACs **59** and **60** satisfactorily degrade the protein of interest. Both PROTACs exhibited selective cytotoxicity dependent on hypoxia (IC_50_ values of 1.09 and 0.61 µM, respectively) and normoxia (IC_50_ values of 1.92 and 1.10 µM, respectively). Furthermore, in hypoxia these PROTACs inhibited tumor migration and induced cancer cells apoptosis. In short, this article validates an innovative and promising strategy to control the action of PROTACs, in which introducing cleavable groups under certain physiological conditions helps to increase selectivity and consequently reduce off-target effects of PROTACs^118^.

More recently, Ding et al. (2024) reported a novel, potent and selective thalidomide-based PROTAC for the induction of FLT3 protein degradation. In vitro studies showed that PROTAC **61** (LWY713) exhibited nanomolar degradation potency (DC_50_ = 0.64 nM, D_max_ = 95%) in FLT3-ITD AML MV4-11 cells. The FLT3 protein degradation induced by PROTAC LWY713 results in a potent cytotoxic effect (IC_50_ value of 1.50 nM), as well as G0/G1 cell cycle arrest and apoptosis of MV4-11 cells. In addition, PROTAC LWY713 was shown to be a promising lead degrader with potent anti-tumor activity in vivo, significantly reducing tumor volume in the MV4-11 cell xenograft model^119^.

##### Two-carbon spacer 4-hydroxythalidomide based PROTACs

**Scheme 10:**
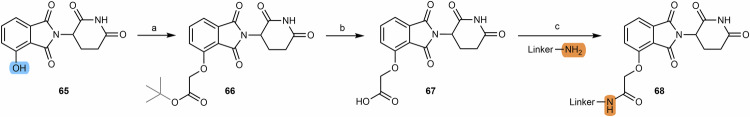
Chemical synthesis of two-carbon spacer 4-hydroxythalidomide based PROTACs. Reagents and conditions: **a**_**1**_ tert-butyl 2- hydroxyacetate, triphenylphosphine (PPh_3_), di-tert-butyl azodicarboxylate (DTBAD), THF, 0 °C to rt, overnight; **a**_**2**_ tert-butyl bromoacetate, potassium iodide, potassium bicarbonate, DMF, 60 °C, 12 h; **a**_**3**_ tert-butyl bromoacetate, potassium bicarbonate, DMF, rt, 2 h; a_4_) benzyl glycolate, triphenylphosphine (PPh_3_), diisopropyl azodicarboxylate (DIAD), THF, 0 °C to rt, 18 h; **b**_**1**_ Formic acid, dichloromethane, 40 °C, overnight; **b**_**2**_ Trifluoroacetic acid, rt, 4 h; **b**_**3**_ Palladium on Carbon (Pd/C), H_2_, methanol, rt, 3 h, quant.; **c**_**1**_ i. Thionyl chloride, DMF or THF, reflux, 4 h; ii. THF, reflux, various times depending on the type of linker (4 h to overnight); **c**_**2**_ HOBT, EDC, triethylamine, THF or DMF, rt, 24 h (time varies depending on the type of linker); **c**_**3**_ HATU, DIPEA, DMF, rt, 24 h (time varies depending on the type of linker).

The synthesis of 4-hydroxythalidomide derivatives with a two-carbon spacer is a strategy that allows obtaining new building blocks to facilitate the construction of cereblon-based PROTACs. By alkylating the 4-hydroxyl group via tert-butyl bromoacetate or benzyl glycolate, followed by removal of the protecting group, it is possible to obtain compound **67**, with a carboxylic acid terminus that can be used for linker attachment via an amide bond, and thus facilitate late-stage linker attachment (Scheme 10)^120–123^. However, it is necessary to consider that the introduction of a new amide bond increases the susceptibility to hydrolysis and the topological polar surface area (TPSA), as well as contributing to the increase of hydrogen bond donor (HBD) or hydrogen bond acceptor (HBA) count.

The alkylation of 4-hydroxythalidomide with tert-butyl bromoacetate to obtain a carboxylic acid derivative in order to facilitate the binding to the amine-terminal linker, for example, was carried out by Li’s group in order to synthesize different cereblon-based PROTACs promoting the degradation of the 20S proteasome subunit β5. All reported PROTACs demonstrated potent anti-proliferative effects against several diverse types of cancer cell lines (IC_50_ values between 0.16 and 86.3 µM), with a pronounced effect on the human pharyngeal squamous cell carcinoma FaDu cell line (IC_50_ values between 0.16 µM and 2.39 µM). Among the various compounds tested, PROTACs **62** (DC_50_ = 0.11 µM, D_max_ = 87%) and **63** (DC_50_ = 0.16 µM, D_max_ = 85%) stand out with the most robust in vitro cytotoxic effect against FaDu and resistant KM3/BTZ cell lines. Furthermore, this PROTAC showed in vivo activity, being well-tolerated. Of note is the ability of these compounds to induce G2/M cell cycle arrest, inhibit cell migration and induce apoptosis. Taken together, these results highlight the potential that these 20S proteasome subunit β5 PROTACs present for the treatment of pharyngeal carcinoma, and for the treatment of multiple myeloma (MM) patients resistant to the bortezomib inhibitor^124^.

#### Alkyl-connected thalidomide-based PROTACs

**Scheme 11:**
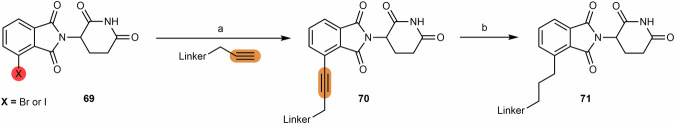
Chemical synthesis of alkyl-connected thalidomide-based PROTACs. Reagents and conditions: **a**_**1**_ (via bromo derivative) Pd(PPh_3_)_2_Cl_2_, Copper (I) iodide, triethylamine, DMF, 70 °C, 3 h; **a**_**2**_ (via bromo derivative) Pd(PPh_3_)_2_Cl_2_, Copper (I) iodide, triethylamine, THF, 70 °C, 12 h; **a**_**3**_ (via iodo derivative) Pd(PPh_3_)_2_Cl_2_, Copper (I) iodide, DIPEA, THF, reflux, 4 to 24 h (time varies depending on the type of linker); **b** Palladium on Carbon (Pd/C), H_2_, ethanol, rt, 12 h.

From the analysis of cereblon-based PROTACs, it is possible to observe a wide variety of alkyl-linked thalidomide derivatives, which include alkine-containing linkers as well as their reduced analogs^110,125^. In this way, it is possible to obtain derivatives with a greater or lesser degree of flexibility or rigidity between the linker and the cereblon ligand. Starting from the iodo- or bromo-thalidomide derivatives **(69)**, it is possible to connect propargyl-containing linkers via Sonogashira coupling reaction **(70)**, with better yields using the bromo derivative (>70%) than the iodo derivative (20–70%)^110,125,126^. The alkyne group of the propargyl-containing thalidomide derivative can be further reduced by Pd/C catalyzed hydrogenation (compound **71**), thereby increasing the degree of flexibility of the linkage (Scheme 11)^110^.

Over the past few years, several alkyl-linked thalidomide-based PROTACs have been published in the scientific literature (Table 7).

**Table 7:** Examples of alkyl-connected thalidomide-based PROTACs

No.	Chemical structure	Target	Biological activity	Ref.
72		PD-L1	*MC-38 cells* *D*_*max*_ > 50% *293 cells* *IC*_*50*_ = 21.18 µM *L-O2 cells* *IC*_*50*_ = 61.01 µM *NIH-3T3 cells* *IC*_*50*_ = 15.65 µM In vivo activity	^127^
73		JAK	*Hel cells* *DC*_*50*_ = 214 nM *D*_*max*_ = 79.5% *IC*_*50*_ = 1.3 µM *IC*_*50*_ = 5.2 nM (enzymatic activity)	^128^

Note:

*JAK* Jakus kinase, *PD-L1* programmed death-ligand 1, *IC₅₀* half-maximal inhibitory concentration, *DC₅₀* half-maximal degradation concentration, *D*_*max*_ half-maximal achievable degradation.

In 2021, Yang’s group reported alkyl-connected PROTAC **72**, targeting programmed cell death-ligand 1 (PD-L1) protein, as a novel cancer immunotherapy strategy^127^. In vitro, PROTAC **72** was shown to effectively degrade the target protein in several cancer cell lines in a time- and concentration-dependent manner (IC_50_ values between 16 and 61 µM)^127^. In vivo studies with PROTAC **72** have demonstrated that by reducing PD-L1 protein levels, it leads to facilitated chemotaxis of CD8^+^ T cells, culminating in significant inhibition of MC-38 tumor growth. Thus, PROTAC **72** has anticancer activity via immune activation^127^.

In 2024, Sheng et al. reported a library of novel JAK1-targeted PROTACs for cancer treatment, using the inhibitor momelotinib (IC_50_ value of 11 nM) as the targeting ligand^128^. Among the various compounds synthesized, the alkyl-based PROTAC **73** stands out, capable of inducing in vitro a selective and potent proteasome-dependent degradation of the target protein (DC_50_ value of 214 nM), resulting in a favorable antiproliferative effect (IC_50_ value of 1.3 µM)^128^. Furthermore, by degrading the JAK1 protein, PROTAC **73** has been shown to reduce the phosphorylation of STAT1 and STAT3, which reinforces the impact of this degrader on the JAK/STAT signaling pathway^128^.

Although several alkyl-linked thalidomide-based PROTACs have been published in scientific literature, it is often observed from SAR studies that a different type of linker linkage favors the degradation activity of the PROTAC and consequently generates better biological activity. This may be due to the lower flexibility of the alkyl-connected thalidomide-based PROTAC, which may not favor the formation of a stable ternary complex essential for the transfer of ubiquitin units from the E3 ligase to the target.

### Lenalidomide-based PROTACs

Lenalidomide is a known ligand of cereblon ligase and is widely used in the construction of cereblon-based PROTACs. Lenalidomide offers advantages over the incorporation of other thalidomide derivatives in the construction of PROTACs, as the absence of a phthalimide carbonyl group confers greater chemical and metabolic stability, better physicochemical properties and lower TPSA^129^. In general, lenalidomide-based PROTACs induce greater degradation of the target protein when compared with their pomalidomide-based counterparts. On the other hand, there are limitations in the synthesis of lenalidomide derivatives (Table 8), since the decrease in the phthalimide ring’s electrophilicity hinders S_N_Ar reactions. Cross-coupling reactions to attach the linker to the lenalidomide derivative can lead to hydrolysis of the cyclic imides^129^.

**Table 8:** Summary of chemical syntheses to obtain lenalidomide derivatives


Substituent (X =)	Linker Connectivity	Reaction name	Conditions	Product
NH_2_, OH	R-X	Alkylation	Base, Δ	
NH_2_	R-COCl	Acylation	Base, Δ	
NH_2_	R-CHO	Reductive amination	NaBH_3_CN, MeOH, Δ	
Br	Ar-B(OR)_2_	Suzuki coupling	Pd(dppf)Cl_2_, base, Δ	
Br	R_1_-NH-R_2_	Buchwald–Hartwitg coupling	Pd-PEPPSI-iHeptCl, Cs_2_CO_3_, dioxane, Δ	
Br, I	R-C ≡ CH	Sonogashira coupling	Pd(PPh_3_)_2_Cl_2_, CuI, base, Δ	
NH_2_	B_2_pin_2_	Miyaura borylation	*t-*BuONO, DBPO, MeCN	
NH_2_	R-SH	Photoredox sulfuration	Ru(bpy)_3_Cl_2_, TsOH, *t-*BuONO	

#### N-alkyl and acyl-connected lenalidomide-based PROTACs

**Scheme 12:**
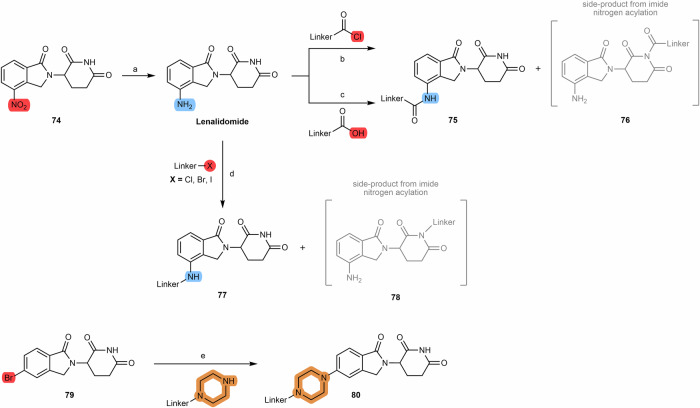
Chemical synthesis of N-alkyl and acyl-connected lenalidomide-based PROTACs. Reagents and conditions: **a**_**1**_ Palladium (II) Hydroxide, H_2_, dioxane, 50 to 60 °C; **a**_**2**_ Fe, Ammonium Chloride, water, ethanol, 80 °C, 4 h; **a**_**3**_ Palladium on Carbon (Pd/C), H_2_, methanol, DMF, quant.; **b** THF, reflux, various times depending on the type of linker (4 h to overnight); **c**_**1**_) Pyridine, phosphoryl chloride, acetonitrile, rt, 3 h (depending on the type of linker); **c**_**2**_ HOBT, EDC, triethylamine, THF or DMF, rt, 24 h (time varies depending on the type of linker); **c**_**3**_ HATU, DIPEA, DMF, rt, 24 h (time varies depending on the type of linker); **d** DIPEA, N-methyl-2-pyrrolidone (NMP), 110 °C, 12 h (depending on the type of linker); **e** Pd-PEPPSI-IHept^Cl^, cesium carbonate, dioxane, 100 °C, 3.5 h (depending on the type of linker).

As described for obtaining pomalidomide derivatives, the amine group of lenalidomide can also be alkylated by alkylhalide linkers or acylated by carboxylic acid linkers (Scheme 12). By reducing the nitro group (e.g., via Pd/C-catalyzed hydrogenation or using Pd(OH)_2_ or iron ammonium chloride) of the starting 2-nitrolenalidomide **(74)**, lenalidomide can be obtained^130,131^. From lenalidomide, it is possible to acylate the 4-amino position of lenalidomide via acid halide or directly with terminal carboxylic acid linkers to obtain acyl-connected lenalidomide PROTACs **(75)**^132,133^. Another option is to derivatize the 4-amino position with chloride, bromine or iodine terminal linkers in DIPEA and using a solvent (e.g., DMSO or NMP) at high temperatures (>90 °C) to obtain N-alkyl-linked lenalidomide PROTACs **(77)**^134–136^. An alternative chemical route is the derivatization of 4- and 5-bromolenalidomide to obtain new lenalidomide derivatives. For example, new 5-aminolenalidomide derivatives **(80)** can be obtained in good yields by Buchwald-Hartwig amination between secondary amine terminal linkers with 5-bromolenalidomide **(79)**^98^. Unlike fluorinated pomalidomide derivatives, S_N_Ar reactions with fluorinated lenalidomide

Several research groups have published different N-alkyl and acyl lenalidomide-based PROTACs (Table 9).

**Table 9:** Examples of N-alkyl and acyl-connected lenalidomide-based PROTACs

No.	Chemical structure	Target	Biological activity	Ref.
81		*EGFR*^*C797S*^	*H1975-TM cells* *DC*_*50*_ = 10.2 nM *D*_*max*_ > 95% *IC*_50_ = 10.3 nM In vivo activity TGI > 50%	^137^
82		ATR	*MV-4-11 cells* *DC*_*50*_ = 22.9 nM *D*_*max*_ = 76% *AML cells* *IC*_*50*_ = 0.11-1.3 µM In vivo activity	^138^
83		BTK	*JeKo-1 cells* *DC*_*50*_ = 7.0 nM *D*_*max*_ = 96.5% In vivo activity	^98^
84		NSD2	*RPMI-8402 cells* *DC*_*50*_ = 20 nM *D*_*max*_ = 96% In vivo activity	^139^

Note:

*ATR* ataxia telangiectasia and Rad3-related protein, *BTK* Bruton’s tyrosine kinase, *EGFR*^*C797S*^ epidermal growth factor receptor C797S mutation, *NSD2* nuclear receptor binding SET domain protein 2, *IC₅₀* half-maximal inhibitory concentration, *DC₅₀* half-maximal degradation concentration, *Dmax* half-maximal achievable degradation, *TGI* tumor growth inhibition concentration.

Yang’s group (2024) published a new anti-EGFR lenalidomide-based PROTAC **81** capable of degrading the EGFR^C797S^ mutant protein for the treatment of lung cancer. PROTAC **81** exhibits nanomolar degradation activity (DC_50_ = 10.2 nM, D_max_ > 95%) against EGFR^C797S^ as well as against EGFR^Del19/T790M/C797S^ mutations, while sparing wild-type EGFR. PROTAC strongly reduces cell proliferation of H1975-TM cells (IC_50_ = 10.3 nM). Oral administration of PROTAC in H1975-TM xenograft tumor model resulted in a significant antitumor effect (TGI > 50%). Since the EGFR^C797S^ mutant protein confers resistance to the inhibitor osimertinib, PROTAC could be a promising alternative treatment for lung cancer patients harboring this mutation^137^.

In 2024, Wang et al. reported a lenalidomide-based PROTAC **82** that potently and selectively degrades ataxia telangiectasia and rad3-related protein (ATR), showing promise for the treatment of AML. Specifically, PROTAC **82** degraded ATR with a DC_50_ value of 22.9 nM and a D_max_ value of 76% in MV-4-11 cells and showed the ability to inhibit cell proliferation of several AML cells (IC_50_ values ranging from 0.11–1.3 µM). PROTAC **82** was also shown to significantly inhibit tumor growth in vivo AML models, outperforming the positive control (VE-821 inhibitor derivative)^138^.

Structurally, both PROTACs **81** and **82** present lenalidomide as a cereblon recruiting moiety linked to the linker via an amide bond^137,138^.

In order to increase the metabolic stability of anti-Bruton’s Tyrosine Kinase (BTK) PROTACs, Wang’s group reported a series of different compounds varying the rigid linker and different cereblon ligands. Among them, the N-alkyl-lenalidomide based PROTAC **83** stands out, inducing the UPS- and concentration-dependent degradation of BTK (DC_50_ = 7.0 nM, D_max_ = 96.5%) without inducing the degradation of cereblon neo-substrates IKZF1/3. In addition to better results in antiproliferative assays compared to the inhibitor ibrutinib, PROTAC **83** showed good metabolic stability with a half-life time of more than 145 min^98^.

In 2024, Liu et al. published a potential and highly selective PROTAC against the nuclear receptor binding SET domain-containing 2 (NSD2) protein, the histone methyltransferase considered a promising cancer target. Existing NSD2 inhibitors are not very selective and not very active. Thus, Liu’s group designed PROTAC **84** (LLC0424), which degrades NSD2 with nanomolar potency (DC_50_ = 20 nM, D_max_ = 96%) in acute lymphoblastic leukemia (ALL) RPMI-8402 cells. Antiproliferative studies demonstrate cytotoxic activity of PROTAC LLC0424 against NSD2-mutant ALL cell lines. In vivo, PROTAC LLC0424 demonstrated potent degradative activity^139^.

#### Alkyl-connected lenalidomide-based PROTACs

**Scheme 13:**
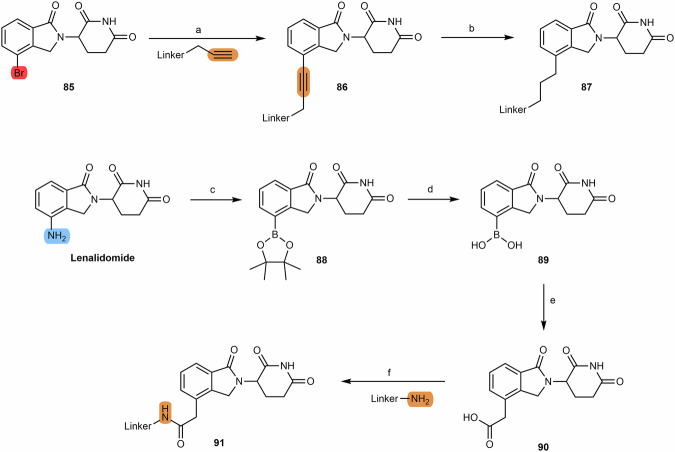
Chemical synthesis of alkyl-connected lenalidomide-based PROTACs. Reagents and conditions: **a** Pd(PPh_3_)_2_Cl_2_, Copper (I) Iodide, triethylamine, DMF, 80 °C, 3 h to overnight (time varies depending on the type of linker); **b**_**1**_ Palladium on Carbon (Pd/C), H_2_, methanol/DMF, rt, 12 h (depending on the type of linker); **b**_**2**_ Palladium on Carbon (Pd/C), H_2_, ethanol, rt, 2 h (depending on the type of linker); **c** t-BuONO, bis(pinacolato)diboron, dibenzoyl peroxide, acetonitrile, rt, 4 h; **d** i. Sodium periodate, THF, water, rt, 2 h; ii. 1 M HCl, rt, 18 h; **e** i. tert-butyl bromoacetate, Pd(PPh_3_)_4_, cesium fluoride, dimethoxyethane, dichloromethane, reflux, 18 h; ii. Trifluoroacetic acid, dichloromethano, rt, 2 h, quant.; **f** HATU, DIPEA, DMF, rt, overnight (depending on the type of linker).

As observed in alkyl-connected thalidomide derivatives, alkyl-lenalidomide derivatives have also been incorporated into the construction of several cereblon-based PROTACs. When establishing the connection between the linker and lenalidomide derivative, an alkyne-type connection is often observed, or an alkane-type connection as a result of the reduction of the previous connection (Scheme 13)^136,140–142^.

Using lenalidomide bromide **(87)** as the starting building block through the Sonogashira cross-coupling reaction, it is possible to perform linker attachment with considerable yields (>40%), depending on the type of alkyne-terminal linkers chosen to incorporate (compound **86**)^143–145^. If the aim is to increase the flexibility of the linker-lenalidomide interface, there is also the possibility of reducing the alkyne-linked derivatives by Pd/C-catalyzed hydrogenation (compound **87**)^146^.

Another strategy for obtaining alkyl lenalidomide derivatives is to convert the amino group of lenalidomide to an aryl boronic ester **88**, which then undergoes oxidative hydrolysis to give compound **89**. Compound **89** can be reacted with tert-butyl bromoacetate via a Suzuki cross-coupling reaction and, after ester hydrolysis, yields a carboxylic acid lenalidomide derivative **(90)** that can be attached to amine-containing linkers, leading to compound **91**^147^.

In 2024, Chen and colleagues reported a series of PROTACs for estrogen receptor (ER) α-positive breast cancer through induction of CDK9 protein degradation. In in vitro studies, cereblon-based PROTAC **92** (L055) was shown to induce potent antiproliferative activity (IC_50_ = 29 nM in MCF-7 cells), G2/M cell cycle arrest, and decreased survival of ERα-positive breast cancer cells. In vivo biological activity evaluation showed that PROTAC L055 reduced tumor growth of T47D and MCF-7 cells in nude mice. All these effects were a result of cereblon-dependent degradation of CDK9 protein promoted by lenalidomide-based PROTAC L055 (Table 10)^148^.

**Table 10:** Examples of alkyl- and alkynyl-connected lenalidomide-based PROTACs

No.	Chemical structure	Target	Biological activity	Ref.
92		CDK9	*MCF-7 cells* *IC*_*50*_ = 29 nM In vivo activity	^148^
93		BTK^C481S^	*Ramos cell line* *DC*_*50*_ = 3.8 nM	^149^
94		STAT5	*AML cells* *DC*_*50*_ = 0.05 to 0.41 µM *D*_*max*_ > 95% *CML cells* *IC*_*50*_ = 0.2 to 2.2 μM In vivo activity	^150^

Note:

*BTK*^*C481S*^ Bruton’s tyrosine kinase C481S mutation, *CDK9* cyclin-dependent kinase 9, *STAT5* signal transducer and activator of transcription 5, *IC₅₀* half-maximal inhibitory concentration, *DC₅₀* half-maximal degradation concentration, *Dmax* half-maximal achievable degradation.

Rao et al. published the lenalidomide-based PROTAC **93** for ibrutinib-resistant malignant lymphoma in 2023. As a result of SAR studies and computational optimization, this group developed PROTAC **93** capable of inducing the degradation of the BTK^C481S^ mutant protein (DC_50_ = 3.8 nM), with a DC_50_ approximately five times higher than that of the first-generation BTK degrader P13I. The cytotoxic effect of PROTAC **93** was also superior to its predecessor in ibrutinib-resistant HBL-1 cells (BTK^C481S^). Another example of a PROTAC capable of overcoming resistance to the use of inhibitors^149^.

In 2023, Kaneshige and co-workers also published a alkyne-connected lenalidomide-based PROTAC for the treatment of AML. PROTAC **94** (AK-2292) targets the signal transducer and activator of transcription 5 (STAT5) protein, a transcriptional factor in the JAK/STAT signaling pathway that regulates tumor cell survival, proliferation, metastasis, and immune evasion. Thus, STAT5 has great therapeutic interest for the treatment of different types of cancer. In vitro studies with a panel of AML cells demonstrated that PROTAC AK-2292 degrades both STAT5A/B isoforms with DC_50_ values ranging from 0.05 to 0.41 µM, and D_max_ values greater than 95%. It is also important to highlight the selectivity of PROTAC AK-2292 for STAT5 over all other STAT proteins. In vivo studies with PROTAC AK-2292 resulted in rapid degradation of the target protein, and consequently in a robust dose-dependent reduction of tumor growth at well-tolerated doses. In another study published by this group, PROTAC AK-2292 demonstrated potent cytotoxic activity against a panel of chronic myeloid leukemia (CML) cells, with IC_50_ values around 0.2 to 2.2 μM, as well as potent antitumor activity in xenograft tumor tissues of human CML NCO2 and KU812 cell lines in mice. Note the complete depletion of STAT5 protein a few hours after administration of the degrader^150^.

#### Phenyl-substituted isoindolinone ligands for PROTAC’s construction

**Scheme 14:**
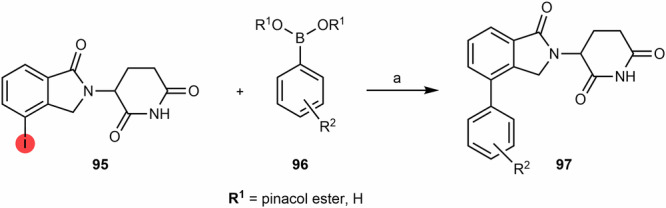
Chemical synthesis of C4-phenyl lenalidomide derivatives. Reagents and conditions: **a** PdCl_2_(dppf) (5 mol%), potassium acetate (3 eq), DMF (0.2 M), 100 °C.

In 2023, Tang and colleagues reported a library of C4-phenyl lenalidomide derivatives resulting from the direct attachment of ortho-, meta- and para-substituted phenyl groups via an optimized Suzuki cross-coupling reaction (Scheme 14)^129^. The authors first synthesize the key iodo-lenalidomide intermediate **(95)** and then replace the iodide by a palladium-catalyzed coupling reaction with a mild base using a boronic ester functional group, isolating the desired products **(97)** using flash column chromatography^129^. In this way, they obtained a series of new lenalidomide derivatives, without verifying the hydrolysis of the cyclic imides. Furthermore, they reported a partial PROTAC library resulting from the ligation of small linkers with terminal benzaldehyde, which can subsequently react with hydrazide group in a 1:1 ratio thus forming acyl-hydrazone linkage PROTACs^129^. Since the only by-product is water, and the solvent is DMSO, the resulting PROTACs can be studied directly in biological studies. Furthermore, if the aim is to improve stability and drug-like properties, the acyl-hydrazone bond can be converted to an amide bond^129^.

In another study, Tang et al. reported new isoindolinone compounds substituted with aromatic rings or saturated six-membered rings^151^. From the analysis of structure-activity relationship studies, it can be concluded that the introduction of a 4-phenyl group contributes to the binding to cereblon. In addition to being cell permeable, these new ligands did not induce degradation of the known cereblon neo-substrate IKZFs^151^.

## Other cereblon-binding scaffolds based PROTACs

Following in vitro and in vivo studies with IMiDs and their respective IMiDs-based PROTACs, their instability has been verified, undergoing hydrolysis and racemization in the majority of culture media used and in body fluids^152,153^. Although the event-driven mechanism of PROTACs may mitigate the impact of compound instability, it can still completely obliterate cellular effects of PROTACs.

**Fig. 4:**
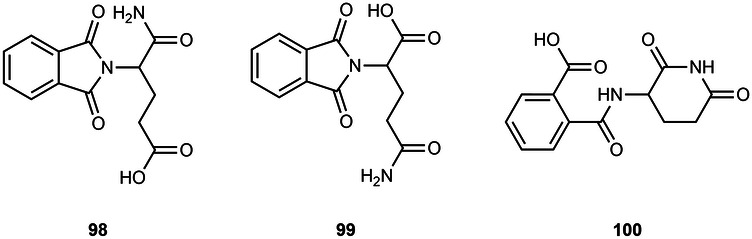
Chemical structure of the compounds resulting from the hydrolysis of thalidomide. The hydrolysis of amide linkages of phthalimido or glutarimide rings of thalidomide essentially forms compounds 98, 99, and 100.

The four carbonyl groups of thalidomide have different pH-dependent susceptibilities to hydrolysis, with the half-life of thalidomide decreasing with increasing pH (pH > 7)^152^. The hydrolysis of amide linkages of phthalimido or glutarimide rings of thalidomide essentially forms products **98**, **99** and **100** (Fig. 4)^152,153^. In addition, an intact glutarimide moiety is often observed in human plasma^154^.

In order to optimize physicochemical properties, increase on-target affinity and reduce instability and off-target neosubstrates, several research groups have developed novel non-phthalimide cereblon binders and incorporated them into the construction of cereblon-based PROTACs.

**Scheme 15:**
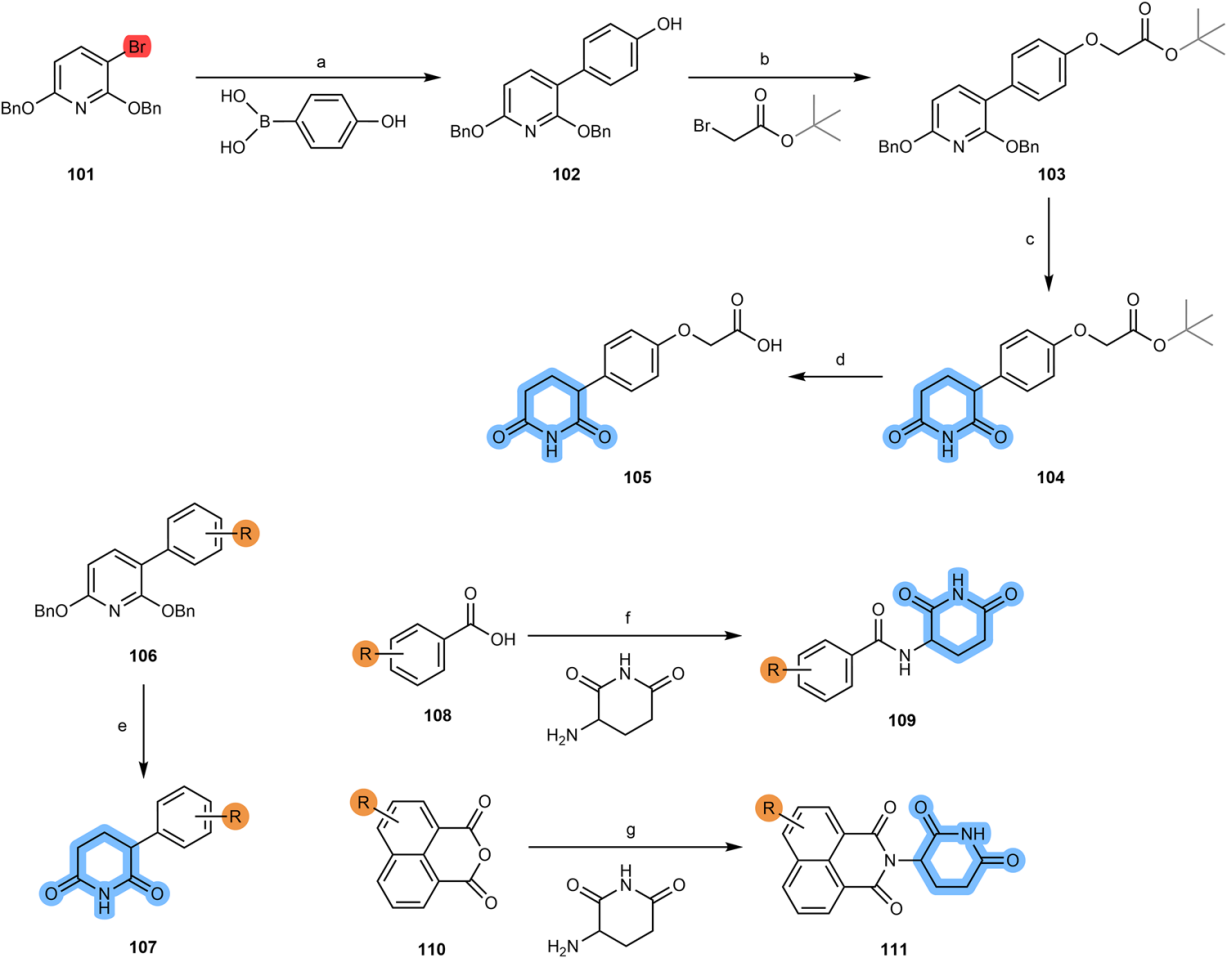
Chemical synthesis of other cereblon-binding scaffolds based PROTACs. Reagents and conditions: **a** PdCl_2_(dppf)×CH_2_Cl_2_, K_3_PO_4_, dioxane/H_2_O, 100 °C, 16 h; **b** K_2_CO_3_, DMF, rt, 3 h; **c** Pd/C, H_2_, EtOH, RT, 16 h; **d** TFA, CH_2_Cl_2_, rt, 2 h; **e**_**1**_ 10% Pd/C, triethylsilane, EtOH, 90 min; **e**_**2**_ H_2_ (15 psi), 10% Pd/C, THF, rt, 4 h; **f** (i) (COCl_2_), DMF (cat.), CH_2_Cl_2_, 0 °C, 2 h; (ii) ArCOCl, Et_3_N, CH_2_Cl_2_, 0 °C to rt, 18 h; **g** THF, 75 °C, 1 h.

To synthesize new PG-based PROTACs, many research groups use the Suzuki cross-coupling reaction between 4-hydroxyphenylboronic acid and compound 101 to synthesize compound 102 (Scheme 15). The hydroxyl group of compound 102 can be alkylated with tert-butyl bromoacetate to give compound 103 which, after hydrogenation of the aromatic ring to glutarimide, gives the PG ligand 104. After removal of the protecting group, the final compound **105** is obtained. It should be noted that the yields obtained in this way are relatively high. To increase the versatility of the linkers for incorporation into cereblon-based PROTACs, it is possible to synthesize or obtain substituted phenyl derivatives (compound **106**) which, after hydrogenation of the aromatic ring, give compound **107**. Depending on the substituent, compound **107** can be attached to different types of linkers to construct novel cereblon-binding scaffolds based on PROTACs. Another strategy to obtain new cereblon ligands consists of coupling 3-aminopiperidine-2,6-dione hydrochloride with compound **108**, via acid halide or directly through coupling agents, thus obtaining different ligands for incorporation in the construction of new cereblon-based PROTACs (compound **109**).

In 2021, Rankovic et al. hypothesized that the electron-withdrawing phthalimide ring might favor the hydrolysis of the glutarimide ring^155^. Therefore, they designed a series of new and smaller cereblon ligands, phenyl glutarimide (PG) analogs. These ligands showed improved chemical stability with similar binding affinity to cereblon^155^. Furthermore, incorporation of these ligands into cereblon-based PROTACs has been shown to increase their potency and degradation efficacy. For example, the PG-based PROTAC **112** (SJ995973) targeting BRD4 protein (DC_50_ value of 0.87 nM) was shown to reduce cell viability in human AML MV4-11 cells at the order of picamolar (IC_50_ value of 3 pM), corroborating the advantages of incorporating PG derivatives in the construction of innovative and more stable cereblon-based PROTACs^155^. Unfortunately, PROTAC SJ995973 demonstrates poor in vivo PK profile (Table 11)^155^.

**Table 11:** Examples of other cereblon-binding scaffolds based PROTACs

No.	Chemical structure	Target	Biological activity	Ref.
112		BRD4	*MV4-11 cells* *DC*_*50*_ = 0.87 nM *IC*_*50*_ = 3 pM In vivo activity	^155^
113		LCK	*KOPT-K1 cells* *DC*_50_ = 0.8 nM *IC*_*50*_ = 37 nM	^156^
114		BRD4	*HEK293 LgBiT stable cells* *DC*_*50*_ = 0.19 nM *D*_*max*_ > 95% *MV4-11 cells* *IC*_*50*_ = 0.12 nM In vivo activity	^157^
115		PD-L1	*4T1 cells* *DC*_*50*_ = 0.609 µM *D*_*max*_ = 85% In vivo activity TGI = 83.23%	^158^
116		BRD4	*MOLT4 cells* *D*_*max*_ > 99%	^159^
117		HCV NS3	*Wild-type NS3 cells (Huh7.5 cells)* *IC*_*50*_ = 748 nM *Cell lines harboring the mutant proteins NS3*^*V55A*^ *and NS3*^*A156S*^ *IC*_50_ (NS3^V55A^) = 508 nM *IC*_*50*_ (NS3^A156S^) = 1561 nM	^58^
118		BCR-ABL	*K562 cells* *D*_*max*_ > 90% *IC*_*50*_ = 68 nM	^133^

Note:

*BRD4* Bromodomain-containing protein 4, *BCR-ABL* breakpoint cluster region-Abelson tyrosine kinase, *HCV NS3* hepatitis C virus non-structural protein 3, *LCK* lymphocyte-specific protein tyrosine kinase, *PD-L1* programmed death-ligand 1, *IC₅₀* half-maximal inhibitory concentration, *DC₅₀* half-maximal degradation concentration, *Dmax* half-maximal achievable degradation, *TGI* tumor growth inhibition concentration.

Although PG-derivatives are more stable compared to IMiDs, they are still susceptible to hydrolysis in culture media, and the C-3 of the glutarimide ring remains configurationally unstable^156^. In this sense, Rankovic and colleagues published new PG derivatives with not only improved chemical stability, but also without the racemization-prone chiral center in PG by replacing the glutarimide C-3 carbon with a nitrogen atom^156^. From the study of new phenyl dihydrouracil (PD)-based PROTACs designed by this group, PD-PROTAC **113** (SJ43489) stands out, capable of degrading the LCK protein (DC_50_ = 0.8 nM) and inhibiting the cell viability of KOPT-K1 cells with nanomolar potency (IC_50_ = 37 nM)^156^.

In order to optimize the physicochemical characteristics of the cereblon ligands for the development of orally bioavailable PROTACs, Rankovic’s group (2024) conducted a comprehensive in vitro/vivo comparative study with different cereblon ligands analogs^157^. This study resulted in the discovery of the orally bioavailable BET-PROTAC **114** (SJ44236), which in addition to having nanomolar degradative activity, presents a superior in vivo pharmacokinetic profile, with high oral bioavailability (45%) and low clearance (8.85 mL/min/kg)^157^.

In 2024, a paper was published by Li’s group reporting a library of novel PG-based PROTACs targeting PD-L1 protein for the treatment of breast cancer^158^. Among the various PROTACs studied, PROTAC **115** stands out, which was shown to be highly potent in 4T1 cells (DC_50_ value of 0.61 μM), inhibiting the growth of these cells, both in vitro and in vivo. From the results obtained in vivo, it is also possible to verify that the reduction in PD-L1 levels facilitates the infiltration of CD4^+^ T cells and CD8^+^ T cells into tumor, culminating in a reduction in tumor growth. This study highlights the construction of new PG-based PROTACs against a protein other than BRD4, demonstrating the versatility of application of these new cereblon ligands in the construction of new cereblon-based PROTACs.

In recent years, Hartmann’s group has reported other types of non-thalidomide cereblon ligands. In 2023, they reported a series of ortho-locked conformationally substituted benzamide derivatives that are more stable and have a reduced ability to recruit off-target neo-substrates^159^. Potent cereblon-based PROTACs targeting BRD4 (PROTAC **116**) were obtained by incorporating benzidamide-based cereblon ligands^159^.

They also reported a series of 2-((hetero)aryl(methyl))thioglutarimides via the thio-Michael addition reaction and corresponding sulfone derivatives formed by oxidation of the sulfur atom, and a serie of spiro-isoxazole based analogs^160,161^. Overall, these ligands showed favorable lipophilicity and good binding affinities to Cereblon. Studies of PROTACs using this type of ligand have not yet been reported^160,161^.

Studies with tricyclic imide-based PROTACs targeting the hepatitis C virus (HCV) protease and the fibroblast growth factor receptor showed a higher affinity of these alternative ligands to cereblon compared to pomalidomide and lenalidomide^58,162^. Furthermore, incorporation of tricyclic imide ligands did not induce degradation of IKZF1/3 neo-substrates^58,162^. The synthesis of new tricyclic imide ligands can be achieved by the reaction of compound **110** with 3-aminopiperidine-2,6-dione hydrochloride, in THF under reflux (Scheme 15)^58,162^.

More specifically, PROTAC **117** against HCV NS3 protein efficiently degraded the target and inhibited proliferation of wild-type NS3 cells (Huh7.5 cells) with an IC_50_ of 748 nM. It also inhibited cell lines harboring the mutant proteins NS3^V55A^ and NS3^A156S^ with IC_50_ values of 508 nM and 1561 nM, respectively^58^.

Strategies such as the creation of photoswitchable PROTACs and PHOtochemically TArgeting Chimeras (PHOTACs) have been developed to control the action of PROTACs in time and space, thereby increasing their selectivity^11^.

Photoswitchable PROTACs have a photoswitchable module (e.g., an azobenzene group) in their linker, and the *cis*- and *trans*-configurations do not have the same activity, changing their configuration quickly and reversibly after exposure to a certain radiation^163^. Thus, the synthesis of cereblon ligands attached to azobenzene photoswitches linkers has been incorporated into the construction of some cereblon-based PROTACs.

There are several examples of azobenzene-based PROTACs. In the case of the anti-leukemic PROTAC **118** against Breakpoint Cluster Region Abelson (BCR-ABL) protein, there is an amide bond linking lenalidomide to the azo group. PROTAC **118** is inactive in the *cis*-configuration, but after irradiation with visible light it changes to the *trans*-configuration and is therefore active. Upon absorption of UV radiation, it reverts to its *cis*-configuration, and thus its inactive state. This strategy increases the precision and selectivity of the treatment and minimizes unwanted side effects^133^.

Another strategy to control the action of PROTACs is the incorporation of caged cereblon ligands, i.e., ligands with photocleavable groups^164,165^. Although different from photoswitchable PROTACs, these types of PHOTACs are not able to bind to E3 ligases when presenting this group and therefore do not induce polyubiquitination of the target protein^164^. However, upon exposure to a specific wavelength capable of cleaving the inactivating group, the PROTAC becomes active, resulting in the forced degradation of the target^165^. The nitrogen of the glutarimide ring of IMiDs has been chosen as the ideal position for the introduction of a photolabile group, such as the nitroveratryloxy-carbonyl (NVOC) or 6-nitropiperonyloxymethyl (NPOM) group^164,165^.

In short, all these different studies demonstrate the relevance of optimizing the ligands of E3 ligases, in this specific case of cereblon E3 ligase, for their incorporation into the construction of more potent, selective, stable PROTACs with the desirable pharmacokinetic properties for oral administration.

## Cereblon-based PROTACs: non-classic synthetic approaches

As seen previously, the classical synthetic routes for obtaining PROTACs are often a time-consuming and a tedious process, with complicated purifications associated with low yields. In this sense, both academia and the pharmaceutical industry have been trying to develop innovative synthetic strategies with a view to speeding up the PROTACs’ discovery.

### Click-chemistry synthesis

The term “click-chemistry” was first coined in 2001 by K. Barry Sharpless to describe a powerful set of fast, modular, efficient, stereospecific, reliable chemical reactions^166^.

In recent years, click-chemistry has been applied exponentially in the field of PROTAC, since the Huisgen copper-catalyzed alkyne azide cycloaddition (CuAAC) click reaction allows a variety of new triazole compounds to be obtained in a simple, rapid and high-yield manner^167^. Copper-catalyzed click-chemistry is performed under mild reaction conditions with stoichiometric amounts of each component (azide and alkyne building block) and with remarkable functional group compatibility^168^.

The debut of click-chemistry in the field of PROTACs occurred in 2017, with the publication of two papers reporting for the first time the use of CuAAC in the PROTAC synthesis^169,170^.

Since then, an increasing number of PROTACs based on triazole fragments have been reported in the literature. This linker typology is the result of the binding of TLM to ELM through click-chemistry reactions, i.e., a copper-catalyzed huisgen 1,3-dipolar cycloaddition reaction between azides and alkyne groups, under mild chemical conditions^167^. In addition to the satisfactory yields obtained, this route also offers the possibility of obtaining an enormous versatility of linkers that can be incorporated into PROTACs.

**Scheme 16:**
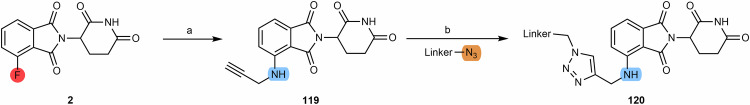
Chemical synthesis of click-chemistry based PROTACs. Reagents and conditions: **a** propargylamine, DIPEA, DMF, 90 °C, 12 h; **b** Copper (II) sulfate, sodium ascorbate, water/tert-butyl alcohol, rt, 16 h (depending on the type of linker).

There are several ways to construct click-chemistry based PROTACs by incorporating cereblon ligands attached to various azide or alkyne terminal linkers. For example, IMiDs derivatives for click-chemistry can be constructed by S_N_Ar reactions between amine-terminal alkine or azide-based linkers and fluorinated thalidomide derivatives, leading to compound **119**^83,132,171^. The final coupling step of the click-chemistry building blocks, i.e., the final coupling of the alkynylated POI ligand derivative with an azide cereblon ligand is accomplished by the addition of sodium ascorbate responsible for catalyzing the reduction of copper (II) sulfate to copper (I) in situ, yielding compound **120**. The click-chemistry reaction can be set up with different solvents, usually in a mixture of solvents such as water and miscible (sometimes only partially miscible) organic solvents such as tert-butyl alcohol or various alcohols (Scheme 16)^125,172,173^.

In click-chemistry based PROTACs, the yield of the reaction depends on the linker to be incorporated (e.g., length, substitutions, binding position to the ligand)^174^. Although the synthesis of triazole-based PROTACs follows the principles of green chemistry, these compounds have low solubilities and are challenging from a pharmaceutical industrial viewpoint due to the difficulty of scale-up.

Several studies indicate that triazole-containing drugs are rapidly metabolized by cytochrome P450 enzymes, and although the metabolism of triazole-based PROTACs has not been extensively studied, it is expected that this may affect their bioavailability and therapeutic efficacy^167,174^.

Given the growing interest in the construction of click-chemistry-based PROTACs, several research groups have reported the preparation of pomalidomide, thalidomide and lenalidomide derivatives for azide-alkyne cycloaddition click reaction.

In 2017, Jung’s group reported the first-in-class triazole-based PROTAC. The cereblon-based PROTAC **121**, targeting sirtuins (Sirt) histone deacetylases, was shown to selectively degrade and inhibit target activity (IC_50_ value of 0.25 µM), thereby inducing downstream α-tubulin hyperacetylation. Thus, this study opened the door to a new strategy for the generation of PROTACs, as well as the generation of PROTACs with potential for the treatment of neurodegenerative diseases, given the involvement of Sirt2 in Parkinson’s disease (Table 12)^169^.

**Table 12:** Examples of cereblon-based PROTACs from non-classic synthetic approaches

No.	Chemical structure	Target	Biological activity	Ref.
121		Sirt	*HeLa cells* *D*_*max*_ > 40% *IC*_*50*_ = 0.25 µM (enzymatic assay)	^169^
122		GSK-3	*PC12 cells* *D*_*max*_ = 65.8% *IC*_*50*_ = 83.1 nM (enzymatic assay)	^175^
123		PD-L1	*A375 cells* *IC*_*50*_ = 8.07 µM *D*_*max*_ > 50%	^176^
124		BRD4	*HiBiT-BRD*_*4*_ *HEK293 CRISPR cells* *DC*_*50*_ = 0.3 nM	^177^

Note:

*BRD4* Bromodomain-containing protein 4, *GSK-3* glycogen synthase kinase 3, *PD-L1* programmed death-ligand 1, *SIRT* sirtuin, *IC₅₀* half-maximal inhibitory concentration, *DC₅₀* half-maximal degradation concentration, *Dmax* half-maximal achievable degradation.

In 2021, Sun and colleagues reported a pioneering library of cereblon-based PROTACs targeting the degradation of glycogen synthase kinase 3 (GSK-3), a protein kinase considered a therapeutic target in neurodegenerative diseases. In vitro studies demonstrated that the triazole-based PROTACs **122** promoted moderate UPS-dependent degradation of POI (D_max_ > 40%), thereby inhibiting glutamate-induced cytotoxicity in HT-22 cells^175^.

Recently, Lie et al. also published a library of novel click-chemistry-based PROTACs recruiting cereblon, MDM2, VHL and IAP E3 ligases for PD-L1 depletion. Among the various compounds, the pomalidomide-based PROTAC **123** stands out with a potent T-cell killing capacity resulting from the forced depletion of the target protein in a dose- and time-dependent manner. This demonstrates its potential as a new strategy for tumor immunotherapy^176^.

### One-pot synthesis

One of the approaches developed to accelerate and simplify the development of new PROTACs and minimize chemical waste by reducing the number of processing and purification steps is one-pot synthesis. The one-pot synthesis strategy consists of combining a target ligand and an E3 ligase ligand with a heterobifunctional linker, under optimized reaction conditions to assemble the desired PROTACs in good yields with minimized by-product formation. This protecting group free strategy allows the multiple reaction steps of PROTACs synthesis to be performed in a single reaction vessel, without the need to isolate intermediates^71,177^.

In 2021, Derksen and co-workers exploited the difference in reactivity between amine nucleophiles to synthesize for the first time pomalidomide-based PROTACs targeting BRD4 through an one-pot synthesis strategy^71^. Thus, they first reacted 4-flurothalidomide with an unprotected heterodiamine linker (NHR-linker-NH_2_), in DMSO at 50 °C to favor the reaction with the secondary amine terminal of the linker. After depletion of 4-flurothalidomide, the activated ester derivative of JQ1 was added at room temperature, which, when reacting with the primary amino terminus of the linker, directly forms the desired compound. By varying the diamine linkers, this group was able to synthesize a library of new pomalidomide-based PROTACs with good yields (20–60%), reducing steps, time, and cost^71^.

However, limitations of this pioneering study include the need to use heteroatom-rich amino linkers and the fact that only nucleophilic substitution reactions work well with thalidomide derivatives, making one-pot synthesis difficult to use with other types of cereblon ligands^177^. In order to expand the toolbox of protocols for one-pot PROTAC synthesis using building blocks with different functional groups, Krumb’s group published a new library of cereblon-based PROTACs targeting BRD4 protein via amide formation after a one-pot synthesis photoinduced C(sp^2^)−C(sp^3^) coupling (yields ranging from 7 to 93%). Various one-pot synthesis-based PROTACs with different aliphatic, aromatic and rigidified heterobifunctional linkers were synthesized, demonstrating the suitability of one-pot transformation for high-throughput screening. When tested in vitro, most of the synthesized anti-BRD4 PROTACs have shown to significantly degrade the target protein, with the most active compound **(124)** showing good solubility and a nanomolar degradational potency (DC_50_ value of 0.3 nM)^177^.

### Multi-Component Reactions (MCRs)

**Fig. 5:**
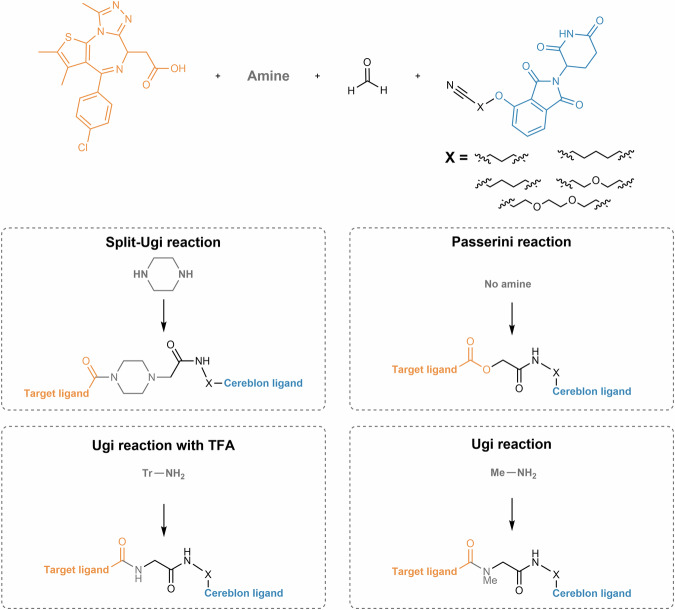
Schematic representation of Multi-Component Reactions (MCRs) for cereblon-based PROTACs. Cereblon-based PROTACs with different linker typologies can be obtained via multicomponent reactions (MCRs), such as, four-component split-Ugi type reaction with a symmetrical diamine, four-component Ugi reaction, and, a three component Passerini coupling reaction.

In 2022, Bhela and co-workers published a paper in which they presented for the first time a multicomponent platform for the synthesis of PROTACs with different linker typologies. These PROTACs were obtained via multicomponent reactions (MCRs) entitled: four-component Ugi reaction, four-component split-Ugi type reaction with a symmetrical diamine and, a three component Passerini coupling (Fig. 5). The advantage of this approach is that through a single one-pot reaction, it is possible to quickly and easily obtain a variety of PROTACs^178^.

In the four-component Ugi reaction, the five different isocyanide derivatives of 4-hydroxythalidomide, in the presence of tritylamine or methylamine, formaldehyde, and acid-containing derivative of the target ligand (JQ1 derivative) yielded different anti-BRD4 PROTACs^178^. In the split-Ugi condensation, the same five isocyanide derivatives underwent reaction with the aldehyde and the acid-ligand, in the presence of piperazine as the symmetric amine^178^. In the Passerini reaction, only the same thalidomide-containing isocyanides with acid and formaldehyde were used. From these four one-pot MCRs, 16 anti-BRD4 PROTACs were obtained, with yields greater than 50%^178^. When tested in MDA-MB-231 cells, MCR-cereblon-based PROTACs were shown to satisfactorily reduce BRD4 levels^178^. From the analysis of SAR studies, it is possible to conclude that PROTACs from the tritylamine Ugi or piperazine split-Ugi condensation gave the highest potency (DC_50_ values between 60 to 240 nM) and highest D_max_ (>86%). On the contrary, PROTACs with tertiary amides from the methylamine Ugi were poorly tolerated and with poor degradational activity^178^.

Therefore, Bhela et al. introduced a novel MCR-based platform designed to facilitate the efficient synthesis of PROTACs^178^. This platform’s great advantage is that it allows extensive exploration of structure-activity relationships by modifying the amine moiety in the MCR process^178^. This involves variations not only in linker length and composition, but also in the point of attachment to the warhead, which has impact on the affinity of the PROTACs and their stability. Importantly, this strategy offers reliability, high yields, versatility, elimination of protecting groups, stereochemical retention, and sustainability.

### Solid-phase synthesis

Solid-phase synthesis (SPS) was invented in the 1960s by Robert Merrifield for the synthesis of peptides^179^. Nowadays, SPS has been a common strategy in synthetic chemistry labs due to its advantages, since through a single filtration of resin-bound chemicals it allows to quickly and simply separate the desired products from the by-products and intermediates existing in the reaction mixture^180^. The drug discovery research area has been taking advantage of the convenient application of SPS in parallel synthesis to create drug-like libraries and in high-throughput chemistry.

**Scheme 17:**
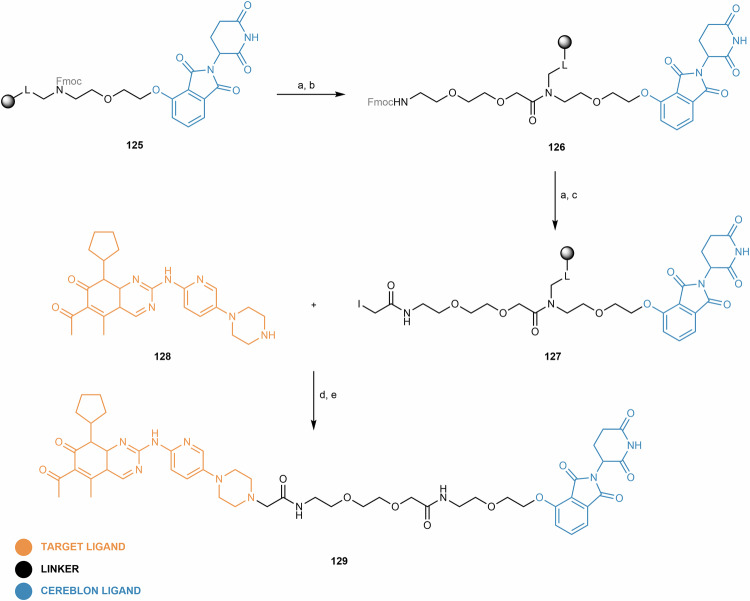
Chemical synthesis of solid-phase based PROTACs (Example 1). Reagents and conditions: **a** DBU/dichloromethane (1:1), rt, 15 min; **b** FAEEAA, HOBt, DIC, DMF/dichloromethane, rt, 16 h; **c** iodocetic acid, DIC, dichloromethane, rt, 90 min; **d** DIPEA, DMSO, rt, 6–16 h; **e** Trifluoroacetic acid/dichloromethane, rt.

In 2019, Krajcovicova’s group pioneered the application of SPS in the discovery of PROTAC. In this paper, the first SPS-based PROTACs were described, which result from a single-step reaction with an on-bead cereblon ligand with kinase inhibitors of CDK4, ALK, BTK, SYK, and EGFR proteins. Thus, on the aminomethyl polystyrene-divinylbenzene (PS-DVB) resin, the link between thalidomide analogs with different linkers was established, forming the final thalidomide-preloaded resin (TPR). After obtaining thalidomide-based electrophiles, they reacted on-bead with five-amine-containing kinase inhibitors via a direct condensation reaction, in the presence of DIPEA. After a final step consisting of an acid treatment, SPS-based cereblon PROTACs are obtained with good yields (Scheme 17)^181^.

**Scheme 18:**
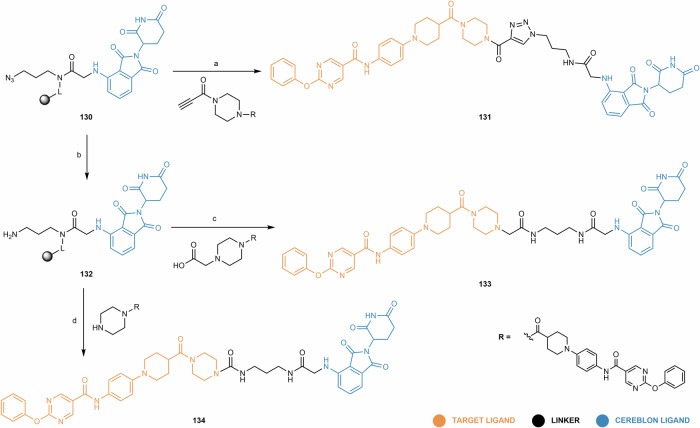
Chemical synthesis of solid-phase based PROTACs (Example 2). Reagents and conditions: **a** (i) Copper (I) iodide, DIPEA, DMF (ii) Trifluoroacetic acid, dichloromethane; **b** THPP, water/DMF; **c** (i) HOBt, HBTU, DIPEA, DMF (ii) Trifluoroacetic acid, dichloromethane; **d** (i) 4-Nitrophenyl chloromata, DMF (ii) Trifluoroacetic acid, dichloromethane.

In 2022, Xu et al. published a series of SPS-based PROTACs targeting hematopoietic prostaglandin D synthase (H-PGDS) and BRD4 proteins resulting from the conjugation of different chemical procedures, such as amide coupling chemistry, click-chemistry, and urea formation. In this work, an intermediate thalidomide-derivatized alkyl azide-resin **130** was first created. From this, the triazole-linked PROTAC was synthesized via azide-alkyne cycloaddition reaction, in the presence of copper (I) iodide (CuI). Amide-linked PROTACs were obtained by reducing the azide group of the resin **130** to the amine group via the Staudinger reaction, subsequently forming the amide linkage with the target ligand. Another option is the formation of urea between the amine of the intermediate and the target ligand using 4-nitrophenyl chloroformate. After the on-bead steps, an acid treatment is performed to cleavage and release the PROTACs with a high degree of purity (>95%). Biological studies with previously synthesized SPS-PROTACs demonstrated that triazole-based PROTACs were the best in degrading H-PGDS protein in KU812 cells. In the case of amide-linked PROTACs targeting the BRD4 protein, they degraded the target protein with nanomolar potency in MV4-11 cells (Scheme 18)^182^.

Although practical examples of SPS-based PROTACs are still scarce and this synthesis method needs to be optimized, the results presented in these studies open doors for easier and faster development of PROTAC libraries targeting different proteins.

### Direct-to-biology (D2B) approaches

The purification of synthesized compounds for biological evaluation is considered the Achilles’ heel of many medicinal chemistry programs, and even more so for PROTACs due to solubility issues. There are a number of strategies that have been published with the aim of speeding up the process of new compound discovery through the reduction of purification requirements. “Direct-to-biology” (D2B) approaches are generally defined as a set of strategies that aim at the biological study of chemical compounds without the need for post-synthesis purification, i.e., biological evaluation of the crude product^183,184^. Although of great interest for high-throughput screening, D2B approaches have strict requirements. Firstly, the synthesis must generate the minimum number of by-products to ensure maximum purity of the final product formed. Second, the reagents and by-products formed should not be toxic or interfere with experimental biological studies. Thirdly, the functional groups of the substrate molecule must be compatible with the reaction conditions.

The first D2B strategy applied to the development of PROTACs was reported in 2020, in which Robert et al. synthesized dozens more hydrazone-linked PROTACs targeting the ER in just a few days, via condensation between aldehydes and acyl-hydrazine ligands in DMSO^184^. After direct evaluation of the crude products (>90% purity by liquid chromatography–mass spectrometry (LCMS), with water as the only by-product), this group resynthesized the best compounds with bioisomeric replacement of the hydrazone by an amide linkage^184^.

Stability problems associated with hydrazone-based PROTACs led Tang’s group to develop the “RAPID-TAC platform” for miniaturized PROTAC synthesis using phthalimidine linkages. Several PROTACs were rapidly synthesized via dialdehyde cyclisation with amine nucleophiles (1:1 ratio in PBS buffer or DMSO at 80 °C), again with water as the sole by-product^185^.

The use of D2B strategies in the development of PROTACs has increased in recent years, particularly in the pharmaceutical industry. In 2022, Janssen published the multistep telescoped anti-BRD4 PROTAC synthesis for accelerated evaluation of linker effects^186^. In this strategy, the central point lies in the choice of mono-N-Boc-protected diamine linkers, followed by a 3-step solid-phase synthetic sequence consisting of an amide coupling, boc-group removal and another amide coupling. In the following year, AstraZeneca also reported the nanomole-scale array synthesis and direct screening of PROTAC reaction mixtures from a pre-assembled library of intermediate E3 ligase ligands attached to different linkers^187^. At the same time, GSK developed an integrated nanoscale D2B-PROTAC platform enabled by automation^188^.

So, by significantly increasing throughput, D2B platforms have gained excellent value and prominence, so research into their use in PROTACs development will undoubtedly continue to evolve.

## Inactive cereblon-based PROTACs

After the discovery that thalidomide derivatives with N-alkylated glutarimide ring are unable to establish binding with cereblon E3 ligase, many research groups began to incorporate these derivatives in the construction of PROTACs with the aim of obtaining negative controls, and thus assessing whether PROTAC-induced degradation is or is not cereblon-dependent^52,189^.

**Scheme 19:**
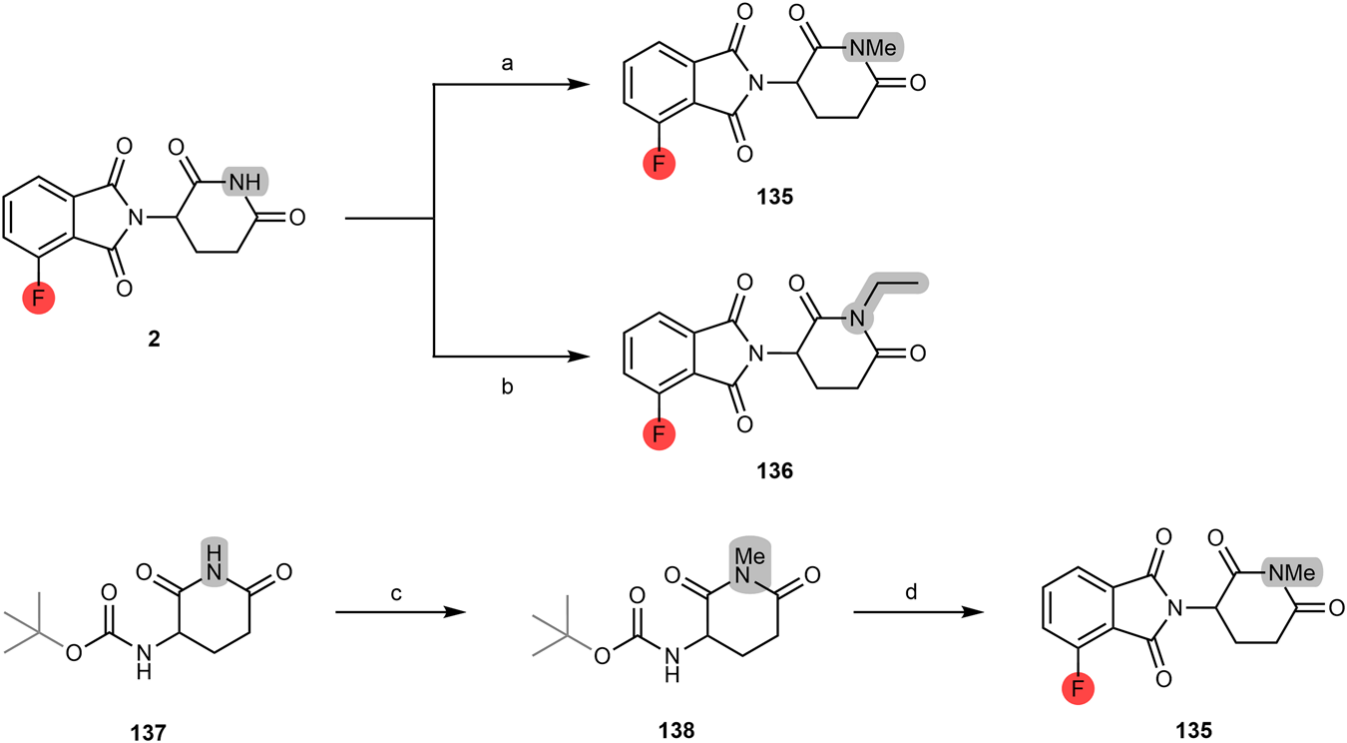
Chemical synthesis of inactive cereblon-based PROTACs. Reagents and conditions: **a** Methanol, Triphenylphosphine (PPh_3_), DIAD, THF, sonication bath, 1 h; **b** Ethyl iodide, potassium carbonate, acetone, reflux, 3 h; **c** Potassium carbonate, methyl iodide, DMF, rt, 2 h; **d** 3-fluorophthalic acid, sodium acetate, acetic acid, reflux, 6 h.

These negative control derivatives can be obtained before or after conjugation of the glutarimide ring moiety to the phthalimide moiety (Scheme 18). In the first case, the alkylation of the imide nitrogen of the glutarimide ring is carried out only after the condensation of the glutarimide and phthalimide moieties, under conditions similar to those of the pre-conjugation alkylation (compounds **135** or **136**)^190^. In the second case, the glutarimide moiety is first alkylated with an alkyl halide **(138)**, such as methyl iodide, in a basic medium, and then the alkylated glutarimide is conjugated with 3-fluoro anhydride to obtain the final 4-fluorothalidomide derivative **(135)**^80,191^ (Scheme 19).

It is still possible to introduce alkyl groups via the Mitsunobu reaction using methanol as the methyl group donor, but the reported yields are lower^80^.

## Cereblon-based PROTACs: factors influencing efficacy

The efficacy of cereblon-based PROTACs depends directly on the absence of mutations that interfere with the binding of the degrader to the protein, or indirectly, on mutations that interfere with the formation of the E3 ubiquitin ligase CRL4^CRBN^ complex, such as mutations that interfere with the binding between cereblon and the DDB1 protein. Over the past few years, results from clinical trials of IMiDs in MM patients have shown that the presence of conferring resistance mutations, loss of gene copy and splice variants of cereblon are associated with resistance to IMiDs and worse clinical outcome^192,193^. For example, patients who are refractory to IMiDs have a higher frequency of cereblon mutations (2–10%) than untreated patients (0.5%). In addition, refractory patients have a higher rate of cereblon gene copy loss (>10%) than untreated patients (1.5%)^194^. The first study to demonstrate in vitro resistance to cereblon-based PROTACs showed that this was due to the downregulation of mRNA and protein, caused by a deletion of a 12 Mbp fragment of chromosome 3 containing the cereblon gene^195^. Another study confirmed that resistance to cereblon-based PROTACs may be caused by a mutation in exon 4 of the cereblon gene, leading to a premature stop codon in the mRNA^196^. Although studies with cereblon-based PROTACs are still scarce, it is expected that long-term clinical use of this type of degrader could lead to the emergence of mutations that prevent PROTAC from binding to cereblon, thus preventing poly-ubiquitination of the target protein.

As mentioned above, IMiDs are indicated for the treatment of MM. However, it is now known that cereblon expression has an impact on therapeutic response, with low expression resulting in a null or weak response to IMiDs^197^. Patients with high cereblon expression showed much better overall survival (OS) and progression-free survival (PFS) in response to IMiDs^198,199^. On the other hand, it is worth noting that the studies by Zhu and co-workers show that treatment with IMiDs leads to a decrease in cereblon expression^200^. Based on the results of previous studies, it is reasonable to assume that IMiDs-based PROTACs may also have their cereblon expression-dependent clinical efficacy. Therefore, depending on the expression level of cereblon protein in the target cell, the therapeutic efficacy of treatment with cereblon-based PROTACs may vary depending on the type of cancer. Long-term treatments with this type of PROTACs may lead to a loss of efficacy, due to a progressive decrease in the expression of this E3 ligase.

The efficacy of cereblon-based PROTACs also depends on the expression of other proteins that are directly or indirectly involved in the ubiquitination of the POI, such as the DDB1 and CUL4 proteins. For example, although cereblon expression is high in some cancers, a lower degradation activity of PROTACs is expected due to the lack of a DDB1/CUL4 protein. In addition, studies of resistance to cereblon-based PROTACs have shown that this may result from downregulation of transcription of E2 ubiquitin conjugating enzyme UBE2G1^196^. Theoretically, cereblon-based PROTACs have the potential to treat diseases in which target cells express prominent levels of not only POI but also Cereblon, DDB1 and CUL4 proteins.

The involvement of efflux proteins is known to be one of the major factors influencing the therapeutic efficacy of drugs, and this is no different in the case of PROTACs. As demonstrated by Kurimchack et al., high endogenous levels of the MDR1 protein resulted in a significant decrease in the efficacy of Cereblon-based PROTAC dBET6 when compared with PROTACs-naïve cancer cells with low expression of MDR1^201^. The use of multidrug resistance protein 1 (MDR1) pump inhibitors restored sensitivity of PROTAC-resistant cancer cells^201^. Analysis of ovarian carcinoma A1847 cells resistant to Cereblon-based PROTACs revealed an upregulation of ABCB1 gene expression^201^. Therefore, it is expected that the upregulation of drug efflux pump(s) of which PROTAC is a substrate will result in a reduction in the amount of degrader that reaches the target cell, and consequently in a lower polyubiquitination of the target protein.

As mentioned above, there are several factors that could cause Cereblon-based PROTACs to become less effective or fail to work. These include reduced expression of cereblon, or other proteins involved in the UPS, genetic alterations including mutations, deletions or alternative splice variants, and upregulation of drug efflux pump(s). However, there are few results on this topic. So, there is a need to explore, identify and understand the factors that may influence the effect of PROTACs, and thus find strategies to optimize their impact.

## Molecular Glue Degraders (MGDs) based on Cereblon ligands

Among the various TPD modalities, molecular glue degraders (MGDs) have gained prominence over the last few years. Similar to PROTACs, MGDs are also an innovative approach that allows inducing forced degradation of a protein of interest^202^. Upon binding of these small molecules to a certain E3 ligase, a conformational change occurs that facilitates the binding of the E3 ligase to the POI (via induction or stabilization of protein-protein interactions), thus culminating in poly-ubiquitination and consequent proteasomal degradation of the target protein^203^. Historically, MGDs have been discovered serendipitously^204^. Most are claimed as inhibitors, but later it is discovered that they generate a neomorphic surface on E3 ligase thus inducing proximity between the E3 ligase and a neo-substrate, leading to the degradation of the latter^205^.

Thalidomide and its derivatives, lenalidomide and pomalidomide, are some of the most well-known MGDs identified so far^203^. As discussed previously, when thalidomide binds to cereblon, it redirects this E3 ligase to the IKZF1/3 proteins, causing their degradation^206^. From the analysis of crystal and cryo-electron microscopy (cryoEM) structural studies it was possible to confirm the role of IMiDs as MGDs, since their binding to cereblon generates a neomorphic surface that confers greater selectivity for a given neo-substrate, resulting from the establishment of IMiD-dependent interactions^51,207^. In addition to inducing the degradation of IKZF1/3 proteins, lenalidomide induces the degradation of the neoabstract casein kinase 1α (CK1α), resulting in its approval for the treatment of myelodysplastic syndrome (MDS)^208^. Pomalidomide additionally degrades the ARID2 neosubstrate^209^. Therefore, several researchers have studied the ability of new chemical modifications in the thalidomide skeleton to induce cereblon-dependent degradation of new neo-substrates.

**Scheme 20:**
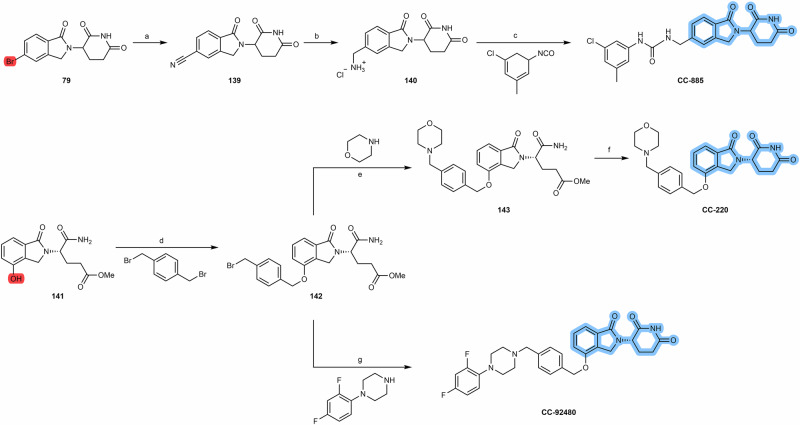
Chemical synthesis of Molecular Glue Degraders (MGDs) based on Cereblon ligands. Reagents and conditions: **a** Pd_2_(dba)_3_, dppf, Zn(CN)_2_, DMF, 120 °C, 19 h; **b** Pd/C, H_2_, HCl, NMP, rt, o.n.; **c** Et_3_N, THF, 40 °C, 5 h; **d** K_2_CO_3_, MeCN, 50 °C, 12 h; **e** CH_2_Cl_2_, rt, 1 h; **f** KOtBu, THF, −78 °C, 30 min; **g** i) KOtBu, THF, −78 °C, 2 h; ii) DIPEA, MeCN, 40 °C, 18 h.

In 2016, the cereblon-based MGD **CC-885** was reported to be capable of inducing the degradation of the transcription factor GSPT1, and thus presenting a potent preclinical anticancer activity, more specifically an antiproliferative effect in AML cells^210^. Through Pd-catalyzed cyanation of 5-bromolenalidomide and consequent reduction of the nitrile group, the Celgene research group obtained intermediate **140** (Scheme 20)^210^. From the reaction of the benzylamine group of intermediate **140** with an isocyanate derivative, compound **CC-885** was subsequently obtained, with good yields^210^.

In 2018, Chamberlain’s group synthesized the new cereblon modulator **CC-220** (Iberdomide), from compound **141**, which when alkylated and subsequently cyclized to form the glutarimide ring, is capable of binding to cereblon with greater affinity than lenalidomide^211,212^. The compound **CC-220** is currently in phase III clinical trials for the treatment of systemic lupus erythematosus and relapsed/refractory multiple myeloma, through the induction of IKZF1 and IKZF3 protein degradation^213^.

Another thalidomide derivative in clinical development is MGD **CC-92480** (Mezigdomide) for the treatment of relapsed refractory multiple myeloma (phase II)^214^. This compound results from an extension, via the piperazine group, of the solvent-exposed side chain in **CC-220**^215^. This structural alteration results in a lenalidomide-based MGD with an increased ability to degrade the IKZF3 protein and thus generate a potent immunostimulatory effect^214,215^.

Compared to PROTACs, MGDs are smaller molecules, with improved drug-like properties, and which allow the degradation of unligandable target proteins, without the occurrence of the hook effect^204^. However, the rational design and synthesis of new MGDs is still little studied. While the number of MGDs reported in scientific literature and in clinical development (approximately 20 MGDs) remains modest, it is anticipated that this domain of TPD will expand in the coming years, driven by a deeper comprehension of the protein-protein interaction interfaces and the more rational development of new compounds^203,204^.

## Cereblon-based PROTACs in clinical trials

According to *clinicaltrials.gov* database, of the approximately 25 PROTACs currently in clinical trials, nineteen recruit the cereblon E3 ligase, the vast majority of which are intended for the treatment of cancer (Table 13). Although much of the clinical trials information on cereblon-based PROTACs is not yet publicly available, it is known that most are intended for oral administration.

**Table 13:** Cereblon-based PROTACs in clinical trials

PROTAC	Chemical Structure	Target	Disease	ROA	NCT number	Phase	Sponsor	Ref.
Vepdegestrant (ARV-471)		ER	Breast cancer	Oral	NCT05654623	III	Arvinas/ Pfizer	^233^
CC-94676		AR	Prostate cancer	Oral	NCT06764485 NCT04428788	III I	Bristol-Myers Squibb	^234,235^
KT-474		IRAK4	Autoimmune diseases	Oral	NCT06058156 NCT06028230	II	Kymera/ Sanofi	^236,237^
Bavdegalutamide (ARV-110)		AR	Prostate cancer	Oral	NCT03888612	I/II	Arvinas	^238^
Luxdegalutamide (ARV-766)		AR	Prostate cancer	Oral	NCT05067140	I/II	Arvinas	^239^
CFT1946		BRAF V600	Solid tumors with BRAF V600 mutations	Oral	NCT05668585	I/II	C4 Therapeutics	^240^
CFT8634		BRD9	Synovial sarcoma and SMARCB1-loss tumors	Oral	NCT05355753	I/II	C4 Therapeutics	^241^
BGB-16673		BTK	B-cell malignancies	Oral	NCT05006716 NCT05294731 NCT06634589	I/II	BeiGene	^242–244^
NX-2127		BTK + IKZF1/3	B-cell malignancies	Oral	NCT04830137	I	Nurix Therapeutics	^245^
FHD-609		BRD9	Synovial sarcoma and SMARCB1-loss tumors	I.V.	NCT04965753	I	Foghorn Therapeutics	^246^
KT-413		IRAK4	B-cell non-Hodgkin lymphoma	I.V.	NCT05233033	I	Kymera Therapeutics	^247^
NX-5948		BTK	B-cell malignancies	Oral	NCT05131022	I	Nurix Therapeutics	^248^
CG001419	Unknown structure	TRK	Solid tumors	-	CTR20222742	II	Cullgen	^223^
BMS-986458	Unknown structure	BCL6	Non-Hodgkin lymphoma	-	NCT06090539	I/II	Bristol-Myers Squibb	^249^
AC682	Unknown structure	ER	Breast cancer	Oral	NCT05080842 NCT05489679	I	Accutar Biotech	^250^
AC676	Unknown structure	BTK	B-cell malignancies	Oral	NCT05780034	I	Accutar Biotech	^251^
HSK29116	Unknown structure	BTK	B-cell malignancies	Oral	NCT04861779	I	Haisco Pharmaceutical Group	^252^
GT-20029	Unknown structure	AR	Androgenetic alopecia and acne vulgaris	Topical	NCT05428449	I	Kintor Pharmaceutical	^253^
HP518	Unknown structure	AR	Prostate cancer	Oral	NCT06155084	I	Hinova Pharmaceuticals	^254^

Note:

*AR* androgen receptor, *BCL6* B-cell lymphoma 6, *BTK* Bruton’s tyrosine kinase, *BRAF V600* B-raf proto-oncogene, serine/threonine kinase V600 mutation, *BRD9* Bromodomain-containing protein 9, *IRAK4* interleukin-1 receptor-associated kinase 4, *ER* estrogen receptor, *IKZF1/3* IKAROS family zinc finger proteins 1/3, *TRK* tropomyosin receptor kinase.

Arvinas, founded by Dr. Crews, is a pioneer company in the commercial development of PROTACs. Currently, it has a strong portfolio of PROTACs, with several degraders already in different phases of clinical trials. The highlights of Arvina’s pipeline are PROTAC **ARV-471** (*Vepdegestrant*), **ARV-110** (*Bavdegalutamide*) and **ARV-766** (*Luxdegalutamide*). PROTAC **ARV-471** is in the most advanced phase of clinical trials (Phase III) for the oral treatment of advanced or metastatic breast cancer driven by ER degradation. In 2024, **ARV-471** received FDA fast-track designation for the treatment of metastatic breast cancer, further strengthening its therapeutic potential^216^. Phase II results showed that PROTAC **ARV-471** reduced ER levels (D_max_ = 89%) and demonstrated a clinical benefit rate of 38% with a good tolerability profile^217,218^. PROTACs **ARV-110** and **ARV-766** are in phase I/II and are intended for the oral treatment of prostate cancer by depleting the AR. Clinical results with PROTAC **ARV-471** demonstrated potent suppression of tumor growth in patients with intrinsic and acquired resistance to enzalutamide (100% and 70%, respectively)^219^. Recently announced clinical data for PROTAC **ARV-766** showed that this degrader is effective in patients with resistance-conferring mutations in the AR gene and has a good tolerability profile^220,221^.

Bristol-Myers Squibb has also invested in the development of new PROTACs, several of which are in clinical trials. Its pipeline includes the cereblon-based PROTAC **CC-94676**, which also targets AR and will enter phase III trials in early 2025. Clinical results published to date show good anti-tumor activity in metastatic castration-resistant prostate cancer (mCRPC) patients with a manageable safety profile^222^.

C4 Therapeutics, Kymera, Nurix Therapeutics, Accutar Biotech are examples of other sponsors with cereblon-based PROTACs in clinical trials, most of which are at an early stage of clinical development (phase I) and therefore clinical results from these trials are still scarce. It should be noted that some of the sponsors to date have partnerships with large pharmaceutical companies (e.g., Pfizer, Sanofi, Novartis or Merck), which highlights the significant funding attracted by this cutting-edge drug strategy, indicating that cereblon-based PROTACs have a huge promising potential.

Given the increasing number of PROTACs recruiting the cereblon E3 ligase in preclinical development, it is expected that the number of these degraders in clinical trials will increase in the coming years.

## Conclusions and future perspectives

PROTACs have revolutionized the entire paradigm of drug discovery and medicinal chemistry research, attracting growing worldwide interest from both academia and the pharmaceutical industry to the extent that articles on PROTACs are published daily. It was the disruptive mechanism of action that made PROTACs, and cereblon-based PROTACs in particular, so successful. By promoting UPS-dependent, non-natural degradation of the target protein, cereblon-based PROTACs have been shown to overcome many of the challenges of occupancy-driven inhibition by SMIs. Further, cereblon-based PROTACs have opened a Pandora’s box by successfully degrading several desirable “undruggable targets” for the treatment of a wide range of diseases^34^.

Having reviewed the main cereblon ligands incorporated in the construction of cereblon-based PROTACs, the synthetic routes for obtaining these degraders, and having analyzed the main results of their biological evaluation, it is still important to discuss and reflect on the current challenges that cereblon-based PROTACs still face, as well as their future prospects.

There are multiple possible synthetic routes to obtain different cereblon ligand derivatives, and subsequent attachment to linkers for the construction of the final cereblon-based PROTACs. The most common chemical reactions to obtain cereblon-based PROTACs include alkylation, acylation, S_N_Ar, Mitsunobu reactions, among others. However, classical synthetic routes are time-consuming, with complicated purifications associated with low yields due to poor chemoselectivity. In order to increase the chemical yield and minimize the formation of by-products, many groups have used strategies to protect the imide groups, such as the introduction *tert*-butyloxycarbonyl (Boc) or p-methoxybenzyl (PMB) as protecting groups, to avoid acylation or alkylation of the nitrogen of the imide ring, which produces inactive compounds^113^. Another strategy is to choose the best binding order of the different building blocks to minimize the formation of by-products under conditions that are not chemoselective for cereblon ligands. In the last few years, new strategies have been applied to accelerate PROTAC screening campaigns in order to obtain cereblon-based PROTACs faster, with high purity and good yields (e.g., click-chemistry, MCRs reactions, D2B approaches).

From the biological evaluation, both in vitro and in vivo, of the many cereblon-based PROTACs reported in recent years, it is worth highlighting that they have several advantageous characteristics, such as: (1) rapid, potent and efficient degradation of POIs, isoforms and protein aggregates; (2) they do not cause overexpression of the target protein or intracellular accumulation of POI; (3) degrade the entire target protein, eliminating enzymatic and non-enzymatic functions; (4) catalytic mechanism of action (event-driven mechanism), reducing the required therapeutic dose and off-target effects; (5) highly selective and specific degradation of POI; (6) degradation of mutant proteins that confer resistance; (7) targeting of “undruggable” protein targets; (8) desirable safety and pharmacokinetic profiles; (9) can be used alone or in combination with other therapeutic strategies. Most of the cereblon-based PROTACs are in preclinical studies, and they have shown very promising therapeutic results not only for the treatment of distinct types of cancer, but also for the treatment of cardiovascular, metabolic, neurodegenerative, immune and infectious diseases. It should also be noted that most PROTACs in clinical trials are cereblon-based PROTACs, with the majority being in phase I.

From the SAR studies, it is possible to verify that the choice of the target ligand, the linker or the cereblon ligand to be incorporated in the construction of cereblon-based PROTACs is not indifferent, nor is the way in which these components are connected to each other. This is because each of these pieces affects not only the degradation activity, but also the selectivity or stability of PROTAC. It is more important to find the best possible combination between the three different building blocks than to look at the effect of each building block individually. Although the information from SAR studies is already considerable, the development of cereblon-based PROTACs is still largely based on trial and error. However, the use of computational tools and artificial intelligence will assist in the rational design of new cereblon-based PROTACs in the near future.

Analysis of the structure of cereblon-based PROTACs shows that the majority incorporate alkyl or intra-oxygenated-based linkers. More recently, the effect of incorporating rigid linkers containing cyclic structures has been studied, as this results in PROTACs that are not only more potent, but also more stable. Nowadays, it is known that the linker is an essential part to obtain the best possible PROTAC, since it influences its geometry and consequently aspects such as solubility, permeability or metabolic stability. In regard to cereblon ligands, derivatives of pomalidomide, thalidomide, and lenalidomide are most commonly incorporated into cereblon-based PROTACs. However, due to instability issues, several research groups have attempted to incorporate more stable and smaller ligands. The future goal is to incorporate better ligands for a robust degradation performance associated with better pharmacokinetic (with good oral bioavailability) and safety profiles, since the choice of ligand affects the ability of the PROTAC to induce degradation of neo-substrates, thus contributing to the occurrence of adverse effects. Therefore, it is highly demanding to optimize all components in order to obtain the best cereblon-based PROTACs.

One of the major challenges in developing novel PROTACs is achieving compounds with favorable oral pharmacokinetic properties. PROTACs typically occupy the beyond-Rule-of-Five (bRo5) chemical space, characterized by high molecular weights (700–1200 Da), elevated lipophilicity, and suboptimal numbers of rotatable bonds (nRotBs), polar surface area, hydrogen bond donors (HBD), and acceptors (HBA)^223,224^. These physicochemical features can adversely affect solubility and permeability, which limits oral absorption and metabolic stability. However, several orally bioavailable PROTACs have been reported, most of them recruiting the cereblon E3 ligase, with some advancing into clinical studies (e.g., ARV-110 or ARV-471). Cereblon-based PROTACs are considered the most ‘oral drug-like’ due to their cereblon ligase ligands. Pomalidomide and lenalidomide, for example, have good oral pharmacokinetic properties, including low molecular weight, acceptable HBD/HBA counts and good lipophilicity. VHL ligands, on the other hand, have a high molecular weight and are bulky^206^. Furthermore, optimizing TLM and linkers can reduce the molecular weight of cereblon-based PROTACs below 700 Da, thereby favoring their oral absorption and metabolic stability. Nevertheless, further research is needed to develop new orally bioavailable cereblon-based PROTACs. Among future strategies, optimizing the molecular properties of cereblon-based PROTACs stands out as a key focus. This involves refining both the target ligands and cereblon ligands - an approach exemplified by the work of Rankovic’s or Tang’s research groups—through the development of smaller, more stable cereblon binders with improved oral pharmacokinetics^129,155,157^. For linker design, the use of shorter, more rigid, all-carbon linkers, as opposed to flexible or PEG-based ones, has been shown to enhance permeability, stability, and reduce efflux susceptibility^225^. Additionally, the integration of machine learning, in silico modeling, and artificial intelligence is expected to accelerate and guide the development of orally bioavailable cereblon-based PROTACs^226,227^.

To date, there are few studies aimed at evaluating the effects of long-term therapy with cereblon-based PROTACs, the factors affecting therapeutic efficacy, and the emergence of resistance to their use. Therefore, results from clinical trials are expected to provide a better understanding of the pharmacokinetics and pharmacodynamics of cereblon-based PROTACs and guide future optimization of their design and development.

The results achieved with cereblon-based PROTACs have opened a new chapter in drug discovery research. The therapeutic possibilities offered by this type of degrader are numerous and relevant. However, a deeper understanding of cereblon-based PROTACs is needed in various aspects, starting from their design and synthesis to their clinical evaluation. Therefore, future efforts are needed to achieve greater rationalization of their design, improvement of their chemical synthesis (less laborious synthetic routes, with fewer steps, good yields, minimal waste and unwanted products), and optimization of their pharmacokinetic/pharmacodynamic profile. In conclusion, although there is still a long way to go and many challenges to overcome, cereblon-based PROTACs have proven to be a very promising and powerful therapeutic weapon against a wide range of diseases.
